# Production of copropophyrin III, biliverdin and bilirubin by the rufomycin producer, *Streptomyces atratus*

**DOI:** 10.3389/fmicb.2023.1092166

**Published:** 2023-03-16

**Authors:** Gustavo Perez-Ortiz, John D. Sidda, Jessica Peate, Davide Ciccarelli, Yaoyu Ding, Sarah M. Barry

**Affiliations:** Department of Chemistry, Faculty of Natural & Mathematical Sciences, King’s College London, London, United Kingdom

**Keywords:** heme, tetrapyrroles, antioxidant, natural product biosynthesis, nitric oxide

## Abstract

Heme is best known for its role as a versatile prosthetic group in prokaryotic and eukaryotic proteins with diverse biological functions including gas and electron transport, as well as a wide array of redox chemistry. However, free heme and related tetrapyrroles also have important roles in the cell. In several bacterial strains, heme biosynthetic precursors and degradation products have been proposed to function as signaling molecules, ion chelators, antioxidants and photoprotectants. While the uptake and degradation of heme by bacterial pathogens is well studied, less is understood about the physiological role of these processes and their products in non-pathogenic bacteria. *Streptomyces* are slow growing soil bacteria known for their extraordinary capacity to produce complex secondary metabolites, particularly many clinically used antibiotics. Here we report the unambiguous identification of three tetrapyrrole metabolites from heme metabolism, coproporphyrin III, biliverdin and bilirubin, in culture extracts of the rufomycin antibiotic producing *Streptomyces atratus* DSM41673. We propose that biliverdin and bilirubin may combat oxidative stress induced by nitric oxide production during rufomycin biosynthesis, and indicate the genes involved in their production. This is, to our knowledge, the first report of the production of all three of these tetrapyrroles by a *Streptomycete*.

## 1. Introduction

Porphyrins underpin many of the key biochemical processes that enable aerobic life (Warren, 2009). Porphyrins are typically enzymatically complexed to transition metals to enable their chemistry, i.e., manganese (chlorophylls), cobalt (vitamin B12), nickel (coenzyme 430), and iron (heme) (Van Norman and Humans, 1974; Bykhovsky et al., 2001; Warren, 2009; Poulos, 2014; Chengjie and Bernhard, 2015). The porphyrin, heme, enables a wide variety of prokaryotic and eukaryotic biochemical processes, from enzymatic transformations, e.g., cytochrome P450 and peroxidase catalysed oxidations, gas transport *via* hemoglobin, to electron transport *via* cytochrome C (Li et al., 2011; Larsen et al., 2012; Poulos, 2014; Fessner, 2019). The fundamental importance of heme in oxygen dependent biochemistry and respiration means that prokaryotes and eukaryotes biosynthesise the cofactor (Dailey et al., 2017). Heme can also have deleterious cellular effects due to its toxicity *via* reactive oxygen species (ROS) production, which some bacterial pathogens have used to their advantage (Quintela-Carvalho et al., 2017). In addition, the hydrophobicity of heme, allows it to partition into the cell membrane and further increase cell susceptibility to reactive oxygen species (Larsen et al., 2012). For these reasons heme biosynthesis must be tightly regulated.

**Figure 1:**
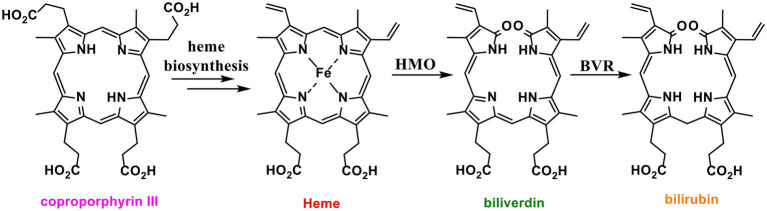
Structures of heme and related tetrapyrroles. Coproporphyrin III is a heme precursor *via* non canonical heme biosynthesis. Heme is degraded to bilirubin *via* biliverdin by the enzymes heme oxygenase (HMO) and biliverdin reductase (BVR).

Most organisms biosynthesise heme *via* one of two pathways. A classical pathway is followed by eukaryotes and Gram-negative bacteria, which biosynthesise heme *via* the key common intermediate protoporphyrinogen IX (Dailey et al., 2017). The non-canonical pathway oxidises the shared intermediate coproporphyrinogen III to coproporphyrin III (Dailey et al., 2015, 2017; Figure 1). Coproporphyrin III is complexed to iron in advance of decarboxylation to produce heme (Dailey et al., 2015). This complex is both the cofactor and substrate for iron-coproporphyrin decarboxylase (HemQ) (Dailey et al., 2010; Milazzo et al., 2018, 2019; Michlits et al., 2020). Thus, unlike the classical heme pathway, the non-canonical pathway produces two iron containing tetrapyrroles (Romao et al., 2000; Ding et al., 2019; Seok et al., 2019).

In microorganisms, coproporphyrin III has been proposed to be involved in the transport of zinc, copper and iron (Yamada-Ankei et al., 1977; Anttila et al., 2011; Azzouzi et al., 2013; Cleary et al., 2018). In fact, proteins have been identified that utilise iron-coproporphyrin III as a prosthetic group instead of heme (Romao et al., 2000). It can also act as an inhibitor of the synthesis of cytochrome oxidase in yeast (Charalampous, 1974). Coproporphyrin III has also been implicated as a chemical signal for interspecies communication. For example, *Propionibacterium* spp. produces coproporphyrin III inducing *Staphylococcus aureus* to aggregate and form biofilms, while in the case of *Glutamicibacter arilaitensis* the production of coproporphyrin III is upregulated by the presence of fungi (Wollenberg et al., 2014; Cleary et al., 2018).

**Figure 2:**
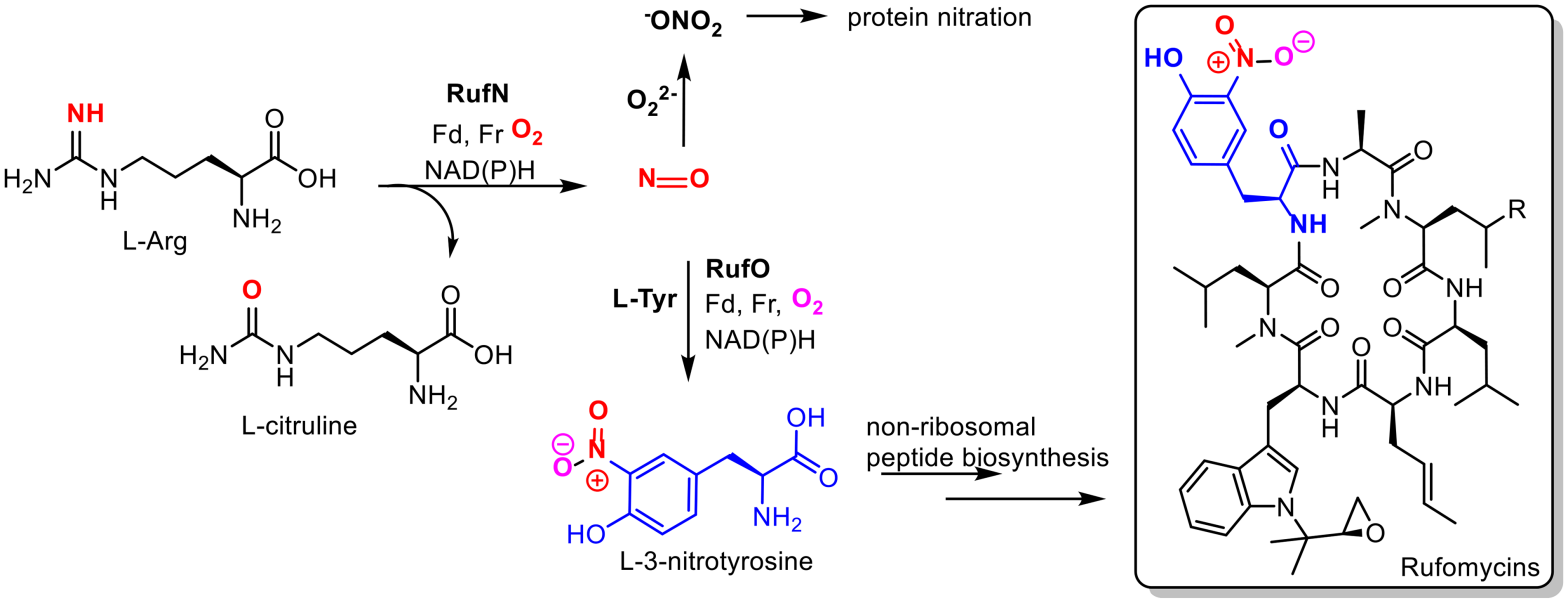
Proposed roles of nitric oxide in *Streptomyces atratus*. Nitric oxide (NO) produced by nitric oxide synthase (RufN) is a biosynthetic precursor used by a cytochrome P450 (RufO) to produce L-3-nitrotyrosine which is incorporated into the non-ribosomal peptide rufomycin by the non-ribosomal peptide synthase, RufT. NO can also react with superoxide to produce reactive nitrogen species, peroxynitrite (^−^ONO_2_), which can damage proteins *via* non-specific nitration of tyrosine and tryptophan residues. (Fd = ferredoxin, Fr= ferredoxin reductase).

Alongside heme biosynthesis, an efficient cellular heme degradation pathway is vital due to the toxicity of heme (Figure 1). Heme oxygenase (HMO) degrades heme to biliverdin, which is then reduced by biliverdin reductase (BVR) to bilirubin (Vítek and Schwertner, 2007; Figure 1). Heme oxygenase has been associated with reducing ROS production (Gozzelino et al., 2010). Heme degradation releases carbon monoxide, biliverdin, and free iron which can contribute to cell damage *via* Fenton chemistry (Jansen and Daiber, 2012). However, biliverdin and bilirubin act as important antioxidants by scavenging reactive oxygen and nitrogen species including nitric oxide (Minetti et al., 1998; Kaur et al., 2003; Vítek and Schwertner, 2007; Wurster et al., 2008). Nitric oxide reacts with oxygen or superoxide to produce reactive nitrogen species (RNS) which can nitrate tryptophan and tyrosine residues, damaging proteins (Figure 2; Radi, 2013). In eukaryotes, *bvr* upregulation has been associated with increased bilirubin production as a major response to oxidative stress (Barañano et al., 2002).

The degradation of heme, and the role of its degradation products, are well studied in eukaryotes but poorly understood in bacteria. In *M. tuberculosis* (*Mtb*) an F_420_H_2_-dependent biliverdin reductase (F-BVR) has been characterised, and bilirubin has been shown to act as an antioxidant and cytoprotectant during the host immune response (Ahmed et al., 2016). *Streptomyces* are, like *Mtb*, actinomycetes, but they are mostly non-pathogenic soil dwelling bacteria. They are a critical genus to drug discovery due to their tremendous capacity for secondary metabolite production, including many clinically used antimicrobials (Hutchings et al., 2019).

While investigating the biosynthesis of the non-ribosomal cyclic peptide, rufomycin, in *Streptomyces atratus,* we analysed the organism’s metabolite profile and identified 3 tetrapyrroles, coproporphyrin III, biliverdin and bilirubin. All were produced under the same growth conditions as those used for rufomycin (Perez Ortiz et al., 2021). Importantly, these conditions do not include any heme supplementation to the growth media. Some *Streptomyces* species have been previously shown to produce coproporphyrin III (Sato et al., 1968; Toriya et al., 1993), but neither biliverdin nor bilirubin have been previously identified in cultures. In *Streptomyces atratus*, nitric oxide (NO) is produced by a nitric oxide synthase (NOS) for the cytochrome P450 catalysed nitration of L-tyrosine. The product 3-nitro-L-tyrosine is a precursor in the biosynthetic pathway of the peptide antibiotic, rufomycin (Tomita et al., 2017; Figure 2). We propose that the production of bilirubin and biliverdin may combat oxidative stress resulting in part from nitric oxide production during rufomycin biosynthesis.

We have analysed the *S. atratus* genome and identified putative genes involved in the biosynthesis of coproporphyrin III and those involved in heme degradation to produce biliverdin and bilirubin. We show that like *Mtb*, the *S. atratus* genome encodes an F_420_H_2_-dependent biliverdin reductase (F-BVR) for production of bilirubin. We also show that this type of BVR, found exclusively in actinobacteria, are found in gene clusters responsible for dealing with oxidative and thermal stress.

## 2. Materials and methods

### 2.1. General methods

Procedures involving bacterial manipulation were conducted in a laminar flow hood (Envair) under sterile conditions. All reagents and solvents used have been stored according to suppliers’ instructions. Coproporphyrin I and III, biliverdin and bilirubin standards were purchased from Sigma Aldrich.

### 2.2. Growth and maintenance of *Streptomyces atratus* DSM41673

*Streptomyces atratus* DSM41673 was purchased as lyophilized mycelia from DSMZ culture collection and grown as instructed. To maintain spore stocks, ISP4 agar plates (Kieser et al., 2000) were inoculated with 20 μl of bacterial spore stocks and incubated at 30°C for 10 days. Sterile water was added to each plate (9 ml to each 25 ml plate), the spores resuspended with a spreader and transferred to a 50 ml falcon tube. Spores were separated by vortex for 1 min and filtered through cotton wool to remove agar. The suspension was centrifuged at 3000 rpm for 5 min. The supernatant was removed, leaving approximately 1,000 μl of water. The spores were resuspended again and an equal volume of 50% glycerol sterile was added to a final concentration of 25% of glycerol. The spore stocks were aliquoted and flash frozen in liquid nitrogen and stored at −80°C.

### 2.3. Production of *Streptomyces atratus* metabolites on solid media

Tetrapyrroles were identified and isolated under the same conditions as rufomycin production and isolation (Perez Ortiz et al., 2021). Briefly, R5 agar plates (Kieser et al., 2000) supplemented with 5 mM L-leucine, were inoculated with 40 μl of *S. atratus* spores and incubated at 30°C for 4–6 days (Media recipe in Supplementary methods). For comparison of metabolite profiles, *S. atratus* was also grown on ISP4 plates and incubated at 30°C for 6 days. In both cases, agar was cut into small squares of approx. 1 cm^2^ and removed from the plates, to be soaked in an equal volume of ethyl acetate, acidified with HCl (pH 2) for 1 h. The ethyl acetate was decanted off and the agar was soaked in acidified ethyl acetate again for another hour. The ethyl acetate solutions were then combined, dried with MgSO_4_, filtered and the solvent removed to produce a dark brown cloudy liquid. The crude extract was stored at −20°C.

To determine the effect of hemin on metabolite profiles a 5 mM hemin (HCL salt, Aldrich) stock was prepared in DMSO, and further diluted to 100 μM prior to being added to the R5 agar plates to a final concentration 10 nM before pouring. These plates were inoculated with 40 μl of *S. atratus* spores and incubated for 6 days at 30°C. To determine the effect of L-NAME on metabolite profiles, a filter sterilized stock solution of L-NAME was prepared in dH_2_O. The solution was added to R5 agar to final concentrations of 1 or 10 μM. These plates were inoculated with *S. atratus* spores and incubated for 4–6 days at 30°C. Crude extracts were prepared for analysis as above.

### 2.4. Isolation and purification of *Streptomyces atratus* metabolites

The crude extract was dissolved in 2 ml HPLC grade acetonitrile and water (50:50) and centrifuged (14,000 rpm) for 10 min. 10 μl of the extract was injected onto the reverse phase Agilent Zorbax C18 column (150 × 4.6 mm, 5 μm particle size) with a flow rate of 0.5 ml min^−1^ (Supplementary Table 1). The detector was set in positive ion mode with a range from *m/z* 200 to 1,300. The UV–Vis absorbance at 222 nm, 282 nm and 355 nm was monitored.

The partial purification of metabolites was carried out using preparative HPLC. The crude extract sample was prepared as for LC–MS. 900 μl of extract was injected onto the C18 reverse phase column (100 × 21.2 mm, 5 μm particle size) at a flow rate of 20 ml min^−1^ (Supplementary Table 2). The UV absorbance of the eluent was monitored at 222 nm, 282 nm and 355 nm. 10 ml fractions were collected (Supplementary Figure 1). The fractions containing the compounds of interest were concentrated by lyophilization. The semi-purified solid was resuspended in 50:50 HPLC grade acetonitrile and water, centrifuged (14,000 rpm for 10 min) and injected onto an analytical reverse phase column Agilent Zorbax C8 column (150 × 4.6 mm, 5 μm particle size) with a flow rate of 0.5 ml min^−1^ to purify the compounds further (Supplementary Table 3). The UV absorbance of the eluent was monitored at 222 nm, 282 nm and 355 nm. Target peaks were collected manually. The solvent was removed by lyophilisation and purified compounds were stored for further analysis at −80°C, covered in foil to protect from photodegradation.

To identify coproporphyrin isomers, the isolated metabolites and standards were dissolved in 50:50 HPLC grade acetonitrile and water, centrifuged (14,000 rpm for 10 min) and injected separately onto an analytical reverse phase column Agilent Zorbax C8 column (150 × 4.6 mm, 5 μm particle size) with a flow rate of 0.5 ml min^−1^ (Supplementary Table 4). The UV absorbance of the eluent was monitored at 280, 355 and 500 nm.

High resolution mass data of biliverdin and coproporphyrin III were acquired using a Waters Acquity UPLC-Class I equipped with an ACQUITY UPLC BEH C8, 1.7 μm, 2.1 × 50 mm column connected to a Waters Xevo-G2-XS QT. Column temperature was 60°C. Buffer A was water +0.1% formic acid, Buffer B was acetonitrile +0.1% formic acid. Flow rate was 0.4 ml min^−1^, with a 1 to 100% Buffer B gradient for 5 min, then 1.5 min of 100% Buffer B (Supplementary Table 5).

The instrument was operated in positive mode full-scan with detection window set from 50 to 700 Da. For product ion scan, a collision energy ramp from 50 to 70 V was employed (Supplementary Figure 2). ^1^H NMR data for coproporphyrin III was recorded on a Bruker 400 MHz NMR instrument in CD_3_OD (Supplementary Figure 3).

### 2.5. Effect of metal ion concentration on *Streptomyces atratus* metabolite production

To study the effect of metal ions from the culture medium on the metabolite profile of *S. atratus*, four different variations of the R5 medium were prepared. R5 media was not supplemented with L-leucine for this part of the study. Standard R5 media was used as control (Kieser et al., 2000). In the other 3 formulas, the concentration of one of the three metals, Cu^2+^, Zn^2+^, and Fe^3+^, respectively, was reduced to 10% of the concentration of the same ion in the control formula. For this, instead of making a solution with all the metals (Trace Elements solution) and adding a volume of this to the final mix, each metal salt was dissolved and sterilized separately and added individually to the final mix (Supplementary methods).

R5 agar plates, with varying trace element concentrations, as described above, were inoculated with 40 μl of *S. atratus* spore stock and incubated at 30°C for 6 days. Each metal ion condition was carried out in triplicate. The metabolites were extracted as described in section 2.3. The crude extract was analysed by LCMS. The area under the curve was used to estimate the amount of each metabolite produced by *S. atratus* in each condition.

### 2.6. Detection of nitric oxide production in *Streptomyces atratus* using fluorescence microscopy

To observe NO production, R5 agar plates were inoculated with *S. atratus* spores and incubated for 5 days at 30°C. One R5 agar plate was treated with the NO synthase inhibitor N-ω-nitro-L-arginine-O-methyl ester hydrochloride (L-NAME) on day 4 of growth (Johnson et al., 2008). A 10 mM stock of L-NAME was prepared in sterile dH_2_O and filter sterilized. This stock solution was diluted to 10 μM in sterile H_2_O and added to the plate and distributed evenly over the mycelia surface with a sterile spreader. The plates were incubated for another 24 h at 30°C.

An area of *S. atratus* mycelia of ~5 mm^2^ on an R5 plate (treated or untreated with L-NAME) was picked from the plate and resuspended in 500 μl sodium phosphate buffer (50 mM, pH 8). 4-Amino-5-Methylamino-2′,7′-Difluorofluorescein Diacetate (DAF-FM DA) dye (5 mM) prepared in DMSO, was diluted to 10 μM in sodium phosphate buffer (Johnson et al., 2008). 500 μl of the dye solution was incubated with the mycelia for 20 min at room temperature. As a negative control, mycelia were treated in the same way, but 500 μl of phosphate buffer was added instead of the dye. Mycelia were allowed to settle, and the dye was removed with a micro pipette. 1 ml of phosphate buffer was added to wash away the excess dye and then fresh buffer was added. The mycelia were incubated in the buffer for 15 min to allow the activation of the dye *via* ester hydrolysis (Supplementary Figure 5). Excess buffer was removed using a micropipette. The mycelia were transferred to an IBIDI-μ-Dish, 35 mm low and allowed to settle to the bottom of the dish before imaging.

The sample was observed on a Nikon A1 confocal on a Nikon Ti-E inverted with a Plan Apo lambda 60×/1.40 Oil magnification. Excitation at 488 nm with a diode laser as a source light, and emission was detected at 515 nm. Images were acquired with a Galvano scanner controlled with NIS elements software. Image analysis was carried out using NIS-Elements. Samples were also observed on widefield microscope Eclipse Ti-2 inverted Nikon microscope (see Supplementary Figure 6).

### 2.7. Bioinformatic analysis of putative genes involved in tetrapyrroles synthesis and metabolism in *Streptomyces atratus*

We used the annotated genes for the non-canonical biosynthetic pathway for the synthesis of the heme group in *S. atratus* SCSIO ZH16 to find the homologs in *S. atratus* DSM41673 using BLASTP (Ma et al., 2017). All the genes show 99.9% identity and are found in the same order and relative location within the genome (data not shown).

We used the biliverdin producing heme oxygenase identified in *S. flavissimus* to perform a BLAST on the *S. atratus* DSM41673 genome (>92.2% identity; Myronovskyi et al., 2013). BLASTP of the surrounding genes was used to annotate the putative functions of the genes, indicating that this gene cluster is involved in heme and iron metabolism.

The gene Rv2074 in *M. tuberculosis*, encodes an F420-depentent biliverdin reductase (F-BVR) (Ahmed et al., 2016). The protein sequence was used to perform BLASTP to identify a similar protein (>48.5% identity) in *S. atratus* DSM41673 (Supplementary Figure 9). A BLASTP of the surrounding genes was used to annotate the putative functions of the genes, indicating that this gene cluster is involved in the respond to oxidative and heat stress. Using each of these proteins as a query, we used tBLASTN to find related proteins in the NCBI Prokaryotic Representative Genomes database.

tBLASTN settings were chosen to only take the top hit for each organism, and the organism’s name, sequence start and end position, strand orientation and protein sequence were collected. Following the tBLASTN searches, the data were combined and filtered to remove duplicates. The identified organisms that did not contain hits for F420-dependent biliverdin reductase were ignored and the top 8 organisms that contained hits for F420-dependent biliverdin reductase were selected for a gene cluster analysis. The genome sequences of the species used in this study are listed in Supplementary Table 6. Individual chromosome sequences with annotations were downloaded from NCBI. The relevant sections of these genomes were aligned using Simple Synteny (Veltri et al., 2016; Supplementary Figure 10). The BLAST E-value, and minimum query coverage cutoff were adjusted to avoid the occurrence of false-positive results.

## 3. Results

### 3.1. Identification of tetrapyrroles coproporphyrin III, biliverdin and bilirubin from *Streptomyces atratus* DSM41673 culture

**Figure 3:**
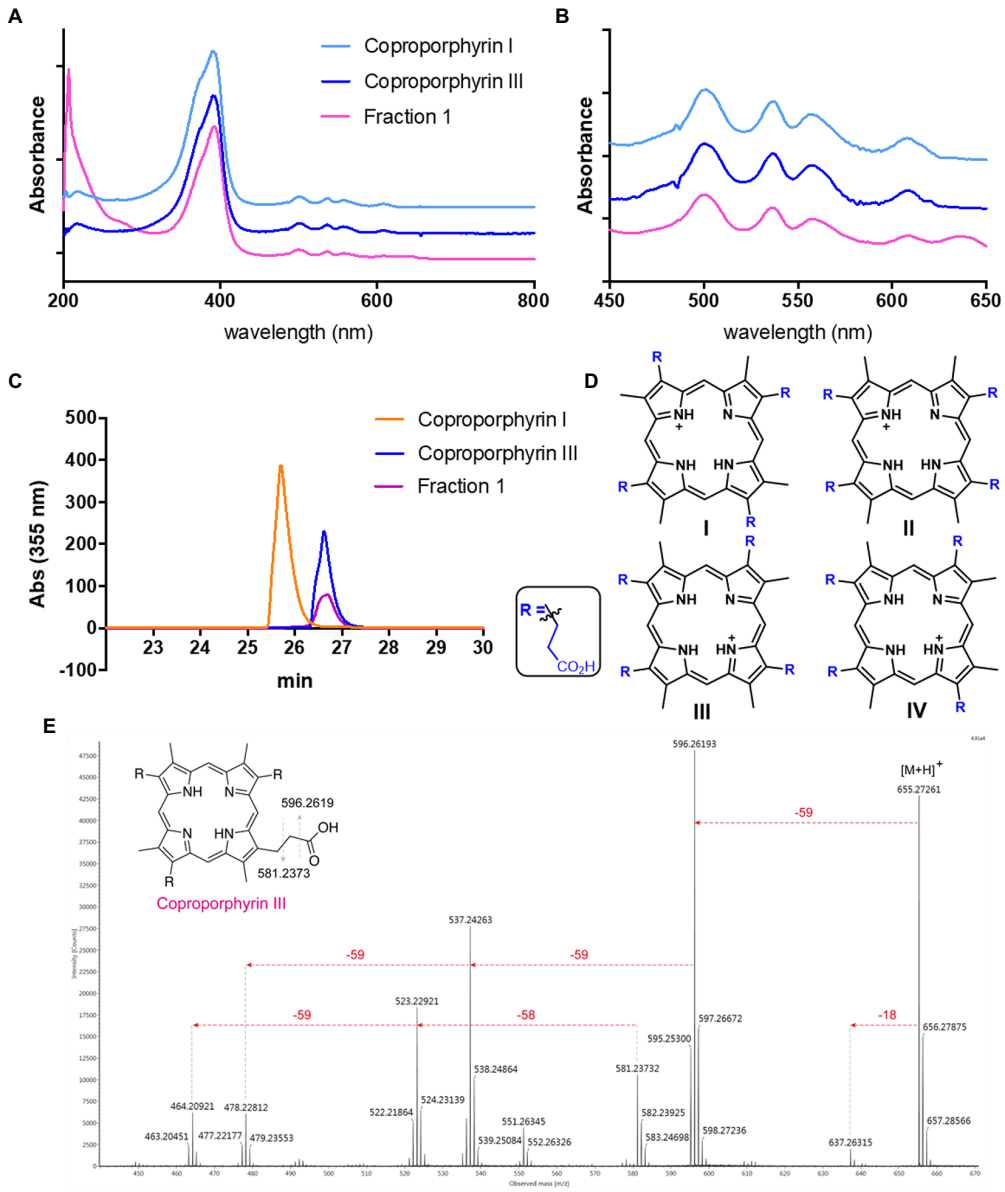
Identification of Coproporphyrin III isolated from *Streptomyces atratus.* **(A)** UV–visible spectrum of tetrapyrrole isolated from *S. atratus* (fraction 1) overlaid with coproporphyrin I & III in 25 mM Tris buffer pH 8. (*λ*_max_ 390 nm). **(B)** Zoom of UV–vis spectra showing Q bands at 500, 536, 557, and 607 nm. **(C)** Analytical HPLC traces at 355 nm of tetrapyrrole isolated from *S. atratus* (Fraction 1) and coproporphyrins I & III standards (Wieland Brown et al., 2013). Retention times of coproporphyrin I and III standards 25.8 and 26.9 min, respectively. (HPLC conditions from Supplementary Table 4). **(D)** Structures of coproporphyrin isomers. **(E)** Fragmentation of 655 m/z ion indicating coproporphyrin species (Supplementary Figure 2). (inset) fragmentation results in [M+H]^+^ fragment ion m/z 596 and radical fragment m/z 581.

*Streptomyces atratus* was cultured on solid R5 media. This defined media is typically used for its ability to induce antibiotic production in *Streptomyces* (Kieser et al., 2000). The agar was extracted following acidification and the crude extract fractionated by preparative HPLC (Perez Ortiz et al., 2021). Two visually striking fractions, one magenta and another green, resulted from the purifications (Supplementary Figure 1). When analysed by LC-HRMS, the magenta fraction (fraction 1) revealed a major peak with a mass of 655.2781 m/z giving a predicted molecular formula of C_36_H_39_N_4_O_8_ and a [M+2H]^2+^ species at 328.1427 m/z (Supplementary Figure 2). Furthermore, the UV–visible spectrum of fraction 1 in 25 mM Tris buffer pH 8, gave a *λ*_max_ of 390 nm and minor peaks at 500, 536, 557, and 607 nm which indicated the presence of a porphyrin like compound (Figures 3A,B; Giovannetti et al., 2004). We compared the UV–vis spectrum of the putative porphyrin in fraction 1 with the spectra of coproporphyrin I and III standards and the result showed identical peaks in the UV–visible spectrum for the three molecules (Figures 3A,B).

Tandem mass spectrometry of the parent ion 655 produced fragments, m/z 637, 596, 537 and 523, corresponding to the reported fragmentation patterns for coproporphyrins (Fateen et al., 2011; Figure 3E). As constitutional coproporphyrin isomers I to IV have the same fragmentation pattern, it is not possible to distinguish constitutional isomers based on tandem MS. Considering that isomers II and IV are rare forms, contributing only 1 and 2% of all coproporphyrins, respectively, the most likely candidates were coproporphyrins I and III (Jacob et al., 1989; Figure 3D). The identity of Fraction 1 was confirmed as coproporphyrin III, using analytical HPLC by comparison with authentic standards of coproporphyrin isomers I and III (Figure 3C). We compared the ^1^H NMR spectra of the compound in fraction 1 and that of the coproporphyrin III standard (Supplementary Figure 3). The spectrum is almost identical, with ppm shifts in several peaks likely due to the presence of the isolated sample as a TFA salt following purification.

**Figure 4:**
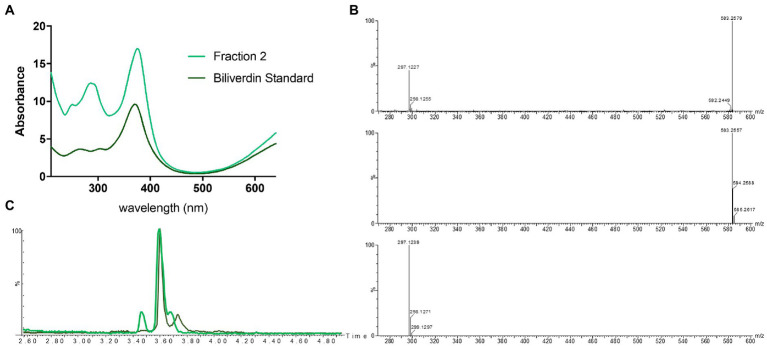
Identification of biliverdin in *Streptomyces atratus* cultures. **(A)** UV–visible spectra of fraction 2 and biliverdin standard in 50:50 water:ACN. **(B)** Mass spectrum of fraction 2 ([M+H]^+^ 583.2579 m/z corresponds to C_33_H_34_N_4_O_6_, error 3.77 ppm) (top) and simulated mass spectrum of biliverdin [M+H]^+^ C_33_H_34_N_4_O_6_ (middle) and and fragment C_17_H_17_N_2_O_3_^+^ (bottom). **(C)** Extracted ion chromatogram ([M+H]^+^ 583.2552 ± 0.01 m/z) of Fraction 2 from *S. atratus* cultures in R5 media (green) and the biliverdin standard (Santoso et al., 2009).

Identification of fraction 1 as coproporphyrin III led us to speculate that the green fraction (fraction 2) may also contain a tetrapyrrole. Analytical LC-HRMS of fraction 2 showed it contained a compound with an [M+H]^+^ of *m/z* 583.2557, which indicated the heme degradation product biliverdin (Figures 1, 4). The retention times and mass spectra of the major components in fraction 2 and the biliverdin standard were identical (Figures 4B,C). The UV-visible spectrum of fraction 2 and the standard showed a *λ*_max_ at 380 nm (Figure 4A). Unfortunately, due to the instability of biliverdin and the small amount isolated, we could not acquire a ^1^H NMR spectrum of sufficient quality. However, based on all other data we conclude that fraction 2 contains biliverdin.

**Figure 5:**
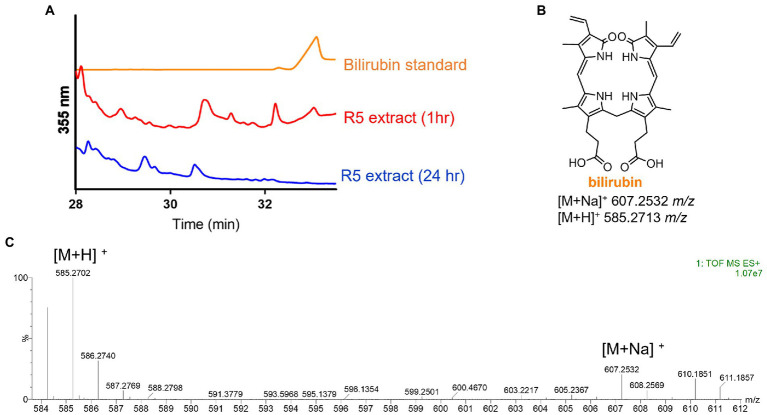
Detection of bilirubin in *Streptomyces atratus* cultures. **(A)** HPLC traces of bilirubin standard and *S. atratus* R5 extract analysed 1 h after extraction and again after 24 h (for low resolution mass spectra of bilirubin peaks, see Supplementary Figure 4). **(B)** Structure of bilirubin and m/z predicted for [M+Na]^+^ C_33_H_36_N_4_NaO_6_ and [M+H]^+^ C_33_H_37_N_4_O_6_. **(C)** High resolution mass spectrum of bilirubin, detected in culture extract within 1hr of extraction.

Having identified the heme degradation product biliverdin, we hypothesized that *S. atratus* may also produce bilirubin *via* reduction of biliverdin (Figure 1). Bilirubin is known to be photolabile and rapidly degrade in aerobic conditions (Ritter et al., 2018). Thus, a fresh crude extract was analysed by LCMS alongside a bilirubin standard. In the fresh extract, a compound with the expected mass and with the same retention time as the bilirubin standard was observed (Figure 5). We were also able to analyse the crude extract by LC-HRMS and confirm molecular formula for this compound (calc. m/z 585.2713, found 585.2702, error − 1.88 ppm; Figure 5; Supplementary Figure 4). This compound was not detected in the sample 24 h after extraction indicating its instability (Figure 5; Supplementary Figure 4).

### 3.2. Genome analysis to identify heme biosynthetic and degradation genes

**Figure 6:**
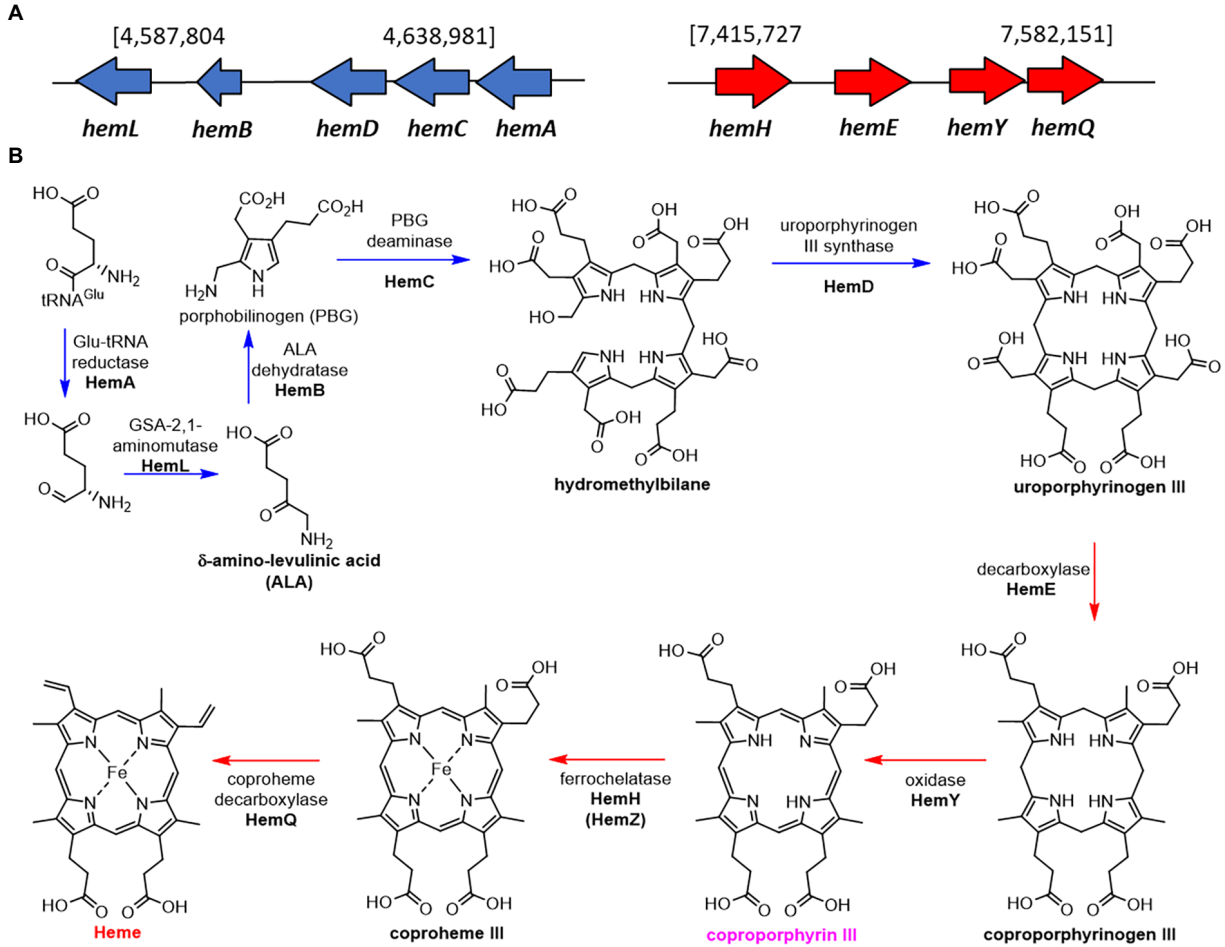
Proposed biosynthetic pathway of heme in *Streptomyces atratus* DSM41673. **(A)** The putative heme biosynthetic genes are separated in two clusters, one containing the genes used for the formation of the porphyrin ring (blue), and a second cluster that ends with the formation of heme *via* the production of coproporphyrin III by HemY (red; Wieland Brown et al., 2013; Supplementary Table 7). **(B)** Proposed heme biosynthetic pathway in *S. atratus via* coproporphyrin III (non-canonical pathway; Choby and Skaar, 2016).

As expected for a Gram -positive bacterium, *S. atratus* produces heme through the noncanonical pathway *via* the production of coproporphyrin III (Choby and Skaar, 2016; Dailey et al., 2017). We identified the putative genes in two clusters, one containing the genes involved in the formation of the porphyrin ring (uroporphyrinogen III), and a second cluster that takes uroporphyrinogen III forms heme *via* the production of coproporphyrin III by HemY (Figure 6).

**Figure 7:**
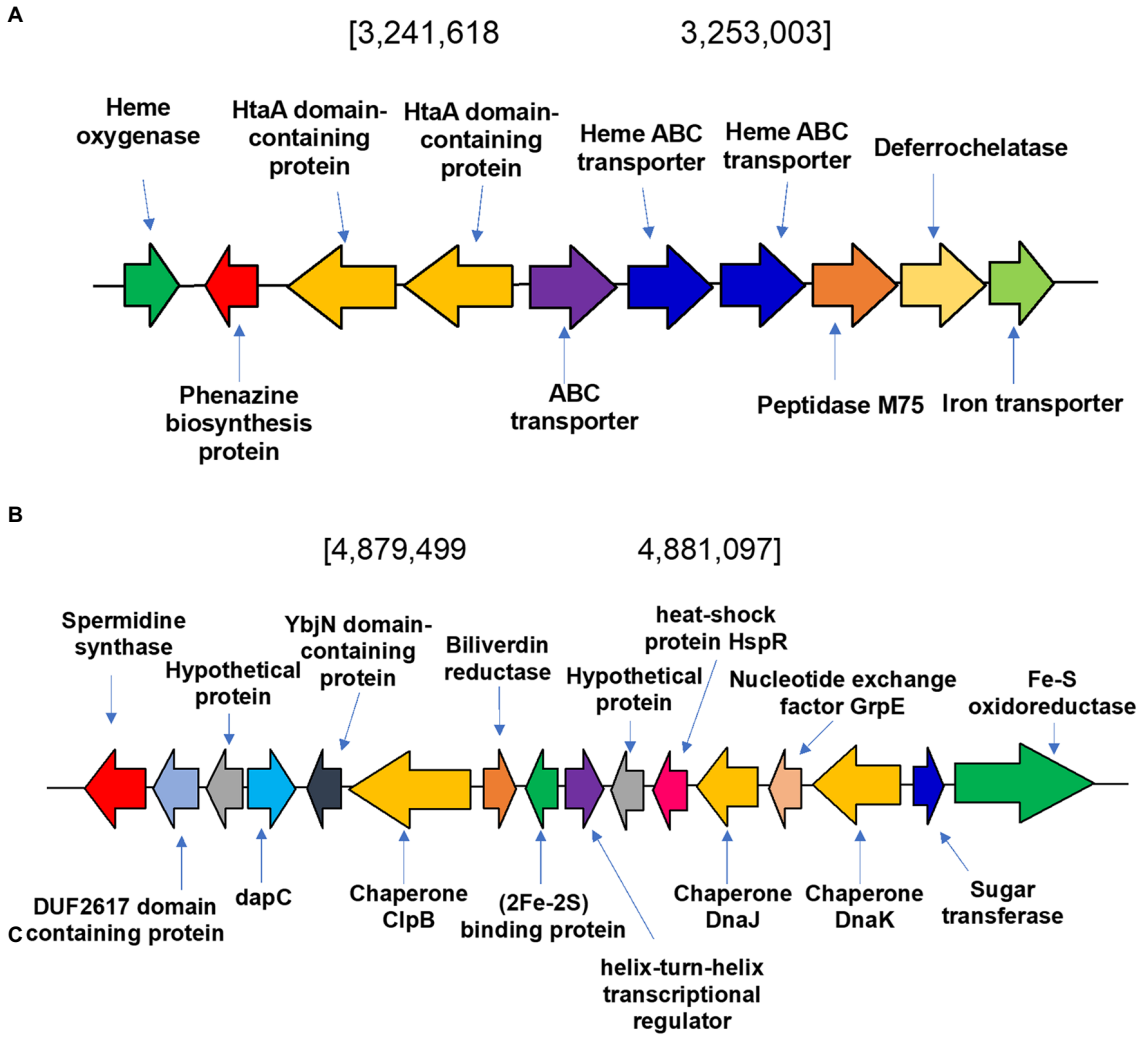
Heme degradation in *Streptomyces atratus* DSM41673. **(A)** Putative heme oxygenase gene is located in a cluster of genes involved in metabolism and transport of heme and iron. In the cluster there are two tandem copies of HtaA domain containing protein and heme ABC transporter, respectively, both involved in heme transport, a putative deferrochelatase and an iron transporter. **(B)** Biliverdin reductase (BVR) is encoded in the *S. atratus* genome in a cluster of genes involved in responding to stimuli such as oxidative stress and heat shock.

To identify heme degradation genes we sought to identify a heme oxygenase (HMO) encoding gene to convert heme to biliverdin and a biliverdin reductase (BVR) encoding gene to produce bilirubin. The heme oxygenase gene (WP_189277514) is in a cluster of genes involved in the metabolism and transport of heme and iron (Figure 7A). This newly identified gene was named *hmo*_satr_. Interestingly, the cluster contains two tandem copies of HtaA domain containing protein and heme ABC transporter, respectively, both involved in heme transport (Uluisik et al., 2017; Delepelaire, 2019). To determine if heme had any effect on the metabolite profile of *S. atratus*, we grew the bacterium on R5 plates supplemented with hemin. We found that only hemin concentrations in the nM range were tolerated by the bacterium, higher concentrations appeared to be toxic and resulted in significantly reduced growth. The resulting metabolite profile differs significantly from that of cultures grown on unsupplemented R5 agar (Supplementary Figures 1, 8). While coproporphyrin III is produced to similar levels, we did not detect any significant increases in biliverdin production (Supplementary Figure 8). The putative biliverdin heme oxygenase in *S. atratus* bears high similarity (>92% identity) to a previously identified homologous protein encoded in the genome of *S. flavissimus*. In fact, we identified this cluster in a range of Streptomycete genomes (Supplementary Table 7; Supplementary Figure 10; Myronovskyi et al., 2013). The presence of a conserved heme oxygenase is unsurprising given its role in eliminating excess potentially cytotoxic heme (Dailey et al., 2017).

The second step of heme degradation is reduction of biliverdin. A biliverdin reductase encoding gene was identified in the genome of *S. atratus* (Figure 7B). This amino acid sequence bears high similarity (>48.5% identity) to the biliverdin reductase (F-BVR) characterised in *M. tuberculosis* (Ahmed et al., 2016; Supplementary Table 7 and Supplementary Figure 9). The enzyme is unusual in its requirement for the rare flavin cofactor F420 (Greening et al., 2016). F420 dependent BVRs are exclusively found in actinobacteria, including Mycobacteria, making them a promising drug targets (Selengut and Haft, 2010). In *S. atratus* DSM41673 the gene is annotated as encoding a F420-dependent enzyme. In another sequenced rufomycin producing *S. atratus* strain, SCSIO_ZH16 (CP027306), this gene is annotated as a nitrilase (AXE78770.1). This newly identified gene was named *bvr*_satr._

We analysed the genes surrounding *bvr*_satr_. In the case of *S. atratus* the gene is located in a cluster of genes involved in responding to stimuli such as oxidative stress and heat shock (Figure 7B). YbjN domain-containing proteins are associated with temperature sensitivity (Wang et al., 2011). Heat shock proteins have also been identified including, putative chaperones DnaJ and ClpB. These are responsible for protecting other proteins from aggregation and misfolding, particularly in stress conditions (Lee et al., 2003; Susin et al., 2006). Next to the biliverdin reductase gene is a gene encoding a putative iron-sulfur cluster protein, annotated as a Reiske oxygenase in many genomes. This arrangement was found to be conserved in many *Streptomyces* strains (examples Supplementary Figure 10) but it differs significantly from the genetic context of *F-bvr* in Mtb (Supplementary Figure 11).

Alignment of the [2Fe-2S] protein from *Streptomyces atratus* with characterised Reiske oxygenases, showed that the protein lacks the non-heme iron domain required for catalysis (Supplementary Figure 12; Kweon et al., 2008). Thus, this gene is misannotated as encoding a Rieske oxygenase and the protein must have another role. This putative iron-sulfur cluster protein also does not contain a conserved Rieske cluster binding motif (**C**X**H**Xn**C**XX**H**). However, there are sufficient histidine and cysteine residues in the protein sequence to complete a (2Fe-2S) cluster (Supplementary Figure 12). Iron–sulfur cluster containing proteins have been shown to be capable of gas sensing, including NO and O_2_ (Mettert and Kiley, 2015). However, BLAST searches and alignments did not indicate any significant similarity between this putative iron–sulfur protein and known NO and O_2_ sensing proteins including NsrR, a known NO detecting protein in *Streptomyces* (Mettert and Kiley, 2015).

### 3.3. Effect of metal ions on coproporphyrin III production

We aimed to investigate the reason *S. atratus*, secretes coproporphyrin III into the medium. Other species of *Streptomyces* have been shown to produce isolatable quantities of coproporphyrin III (Toriya et al., 1993). There are publications that report that several strains of bacteria and fungi produce coproporphyrin III to capture metals such as copper and zinc from their environment (Azzouzi et al., 2013; Cleary et al., 2018). This porphyrin also binds iron, and there proteins that have iron-coproporphyrin III as a prosthetic group (Romao et al., 2000).

To determine if *S. atratus* uses coproporphyrin III as a metal chelator, *S. atratus* was grown on agar where one of the metal salts in R5 was reduced to 10% of the original concentration (4 mg/L ZnCl_2_, 20 mg/L FeCl_3_•6H_2_O, 1 mg/L CuCl_2_•2H_2_O, respectively). *Streptomyces atratus* was grown on these plates as usual for 6 days. The cell growth was not affected in any experiment. We reasoned that if the role of coproporphyrin III was to capture ions from the medium, reducing the concentration of ions would induce the cells to produce more coproporphyrin to improve the absorption of this ion.

**Figure 8:**
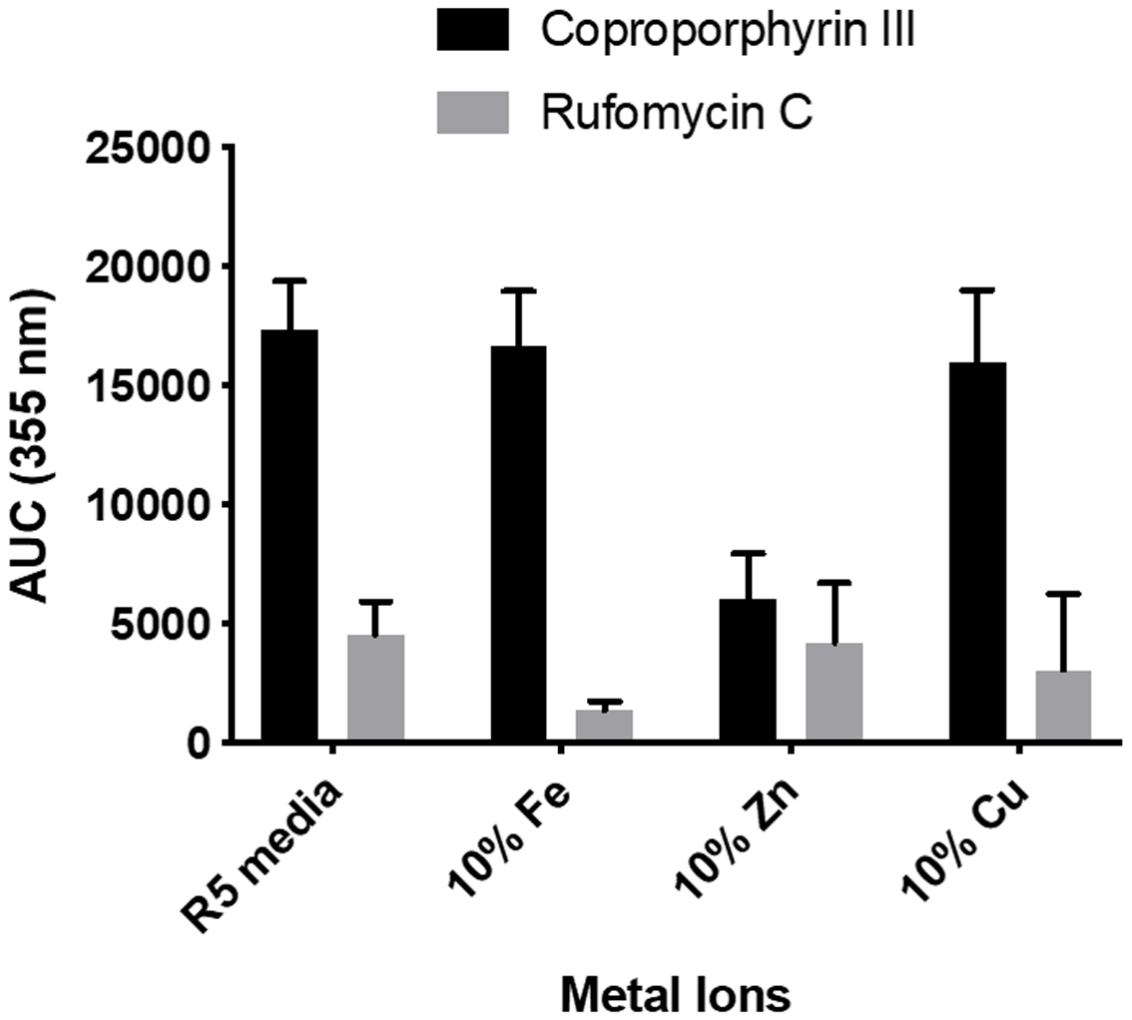
Effect of reduced metal ion concentration on coproporphyrin III and rufomycin production. R5 media contains 0.04 mg/L of Fe^3+^, 0.02 mg/L of Zn^2+^, and 0.004 mg/L of Cu^2+^. For reduced metal concentrations only the named metal concentration was changed, all other metals were added as per the control. Plates were incubated at 30°C for 6 days. Extracts were analysed by LCMS and the AUC of the peaks at *λ*_max_ 355 nm was calculated. Each data point is an average of 3 replicates.

We observed no changes in the concentration of coproporphyrin III produced when Fe^3+^ or Cu^2+^ concentrations were reduced. Interestingly, in the case of the low zinc medium, the production of coproporphyrin III decreased considerably (Figure 8). Heme biosynthesis enzyme, HemB, is a zinc dependent protein and thus production of coproporphyrin III maybe affected in a low zinc medium (Chauhan et al., 1997). We also measured the effect on rufomycin production. As heme is important for rufomycin production [there are five heme dependent biosynthetic enzymes (Ma et al., 2017; Tomita et al., 2017; Perez Ortiz et al., 2021)] lower iron in the medium may explain the decrease in rufomycin production. However, interestingly rufomycin production was not affected by reduced zinc or copper.

### 3.4. Fluorescence detection of nitric oxide production

Based on our analysis of the *S. atratus* genome, we hypothesized that nitric oxide production during rufomycin biosynthesis may lead to oxidative stress in *S. atratus* and thus the production of biliverdin and bilirubin as antioxidants. We thus aimed to detect NO production in living *S. atratus* cells using fluorescence microscopy and an NO specific dye. The production of NO by *Streptomyces* has been observed previously in the plant pathogen *S. scabies* which also utilizes NO as a biosynthetic precursor (Johnson et al., 2008; Barry et al., 2012). NO was detected in *S. scabies* cultures, and was proposed to act additionally as a signaling molecule in its interactions with plants (Johnson et al., 2008). *Streptomyces atratus* is not a plant pathogen however it has been identified as a potential biocontrol agent (Liang et al., 2016; Jung et al., 2018).

**Figure 9:**
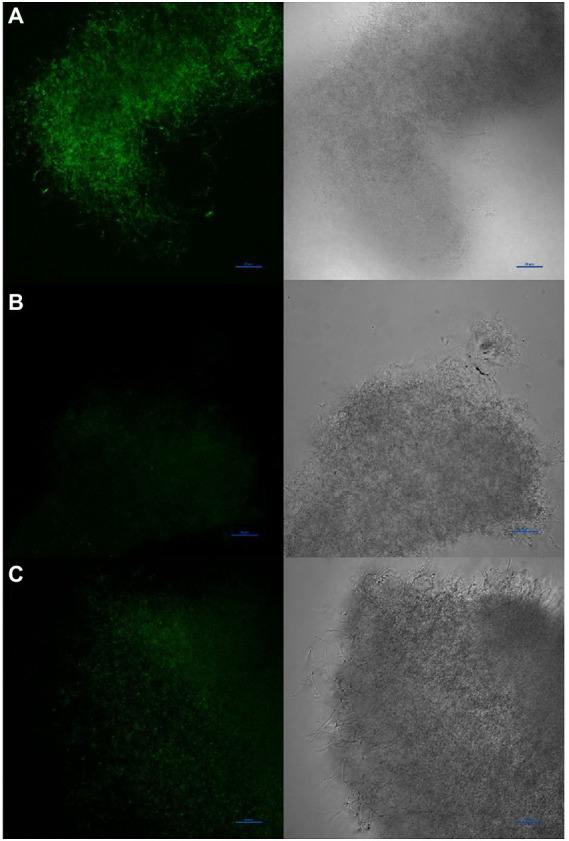
Visualisation of NO production by *Streptomyces atratus* with DAF-FM DA using confocal fluorescence microscopy (Excitation at 488 nm, emission 515 nm). **(A)** mycelia stained with DAF-FM DA, **(B)** unstained control, **(C)** mycelia treated for 24 h with the NOS inhibitor L-NAME before staining. Scale bars are 20 μm.

To determine if diffusible nitric oxide was indeed produced by *S. atratus*, we grew bacteria on R5 media under conditions used for rufomycin and thus NO production. The selective and highly sensitive NO fluorescent dye DAF-FM DA (Supplementary Figure 5) was incubated with the bacterium and used to image the intercellular NO present in the bacterium (Figure 9). DAF-FM DA is not fluorescent but is cell permeable (Kojima and Nagano, 2000). Once it enters the bacterial membrane it is hydrolysed by esterases to form DAF-FM which reacts with low concentrations of intercellular NO to from a benzotriazole fluorescent derivative (Supplementary Figure 5). Some areas of mycelia show concentrated areas of fluorescence suggesting different cellular environments may promote or suppress the production of NO (Figure 9 and Supplementary Figure 6). Additionally, to ensure the NO detected originated from NOS activity, *Streptomyces atratus* was grown on R5 media and treated with the known NOS inhibitor L-NAME. These samples resulted in suppressed fluorescence indicating inhibition of NOS activity and suppressed NO production. Additionally, we investigated the effect of L-NAME on both rufomycin and tetrapyrrole production (Supplementary Figure 7). Analysing crude extracts of *S. atratus* grown on R5 supplemented with L-NAME showed significant (up to 50%) decreases in rufomycin production, which corresponds nicely with inhibition of the NOS, RufN (Figure 2; Supplementary Figure 7A) and thus decreased availability of a key biosynthetic precursor. As expected, coproporphyrin III production was unaffected by the inhibitor. This indicates that coproporphyrin III production is not linked to NOS activity (Supplementary Figure 7B). The production of biliverdin does appear to be reduced in the presence of L-NAME, as expected if its role is to combat NO induced oxidative stress (Supplementary Figure 7D). However, the low levels produced and instability of this compound meant this data was inconclusive using this method. We do note however that biliverdin production increases over incubation time. Only trace levels are detected at 4 days (Supplementary Figure 7C).

## 4. Discussion

*Streptomycetes* are slow growing, soil dwelling bacteria with complex life cycles including a mycelial saprophytic lifestyle and a sporulation phase (Jones and Elliot, 2018). The genus is best known as a crucial source of biologically active secondary metabolites including antibiotics and anticancer agents (Hutchings et al., 2019). The expression of secondary metabolite gene clusters is often associated with starvation conditions due to competition for resources and thus under lab conditions strains are often grown in minimal or defined media to replicate this state. Biosynthesis of many metabolites is induced by the presence or absence of certain metabolites, e.g., sugars, trace elements or “turned on” by quorum sensing signalling molecules (Kweon et al., 2008). The biosynthesis of natural products is also influenced by the flux of primary metabolites, which can affect the availability of pathway precursors to produce cofactors associated with biosynthetic enzymes.

*Streptomyces atratus* produces the nitrated natural product antibiotic peptides, rufomycins. While investigating the rufomycin biosynthetic pathway, we identified three tetrapyrroles from *S. atratus* cultures which are biosynthetic intermediates (coproporphyrin III) or degradation products (biliverdin, bilirubin) of heme. Our analysis of the *S. atratus* genome, indicates that the heme cofactor is biosynthesised *via* the non-canonical pathway known for *Streptomyces*. This pathway proceeds *via* coproporphyrin III (Figure 6).

Coproporphyrin III has been proposed to play a role in metal sequestration and has been isolated in complex with Zn^2+^ (zincphyrin; Anttila et al., 2011; Cleary et al., 2018). Our data shows that reducing the concentration of metal salts in the growth media does not increase coproporphyrin III production, thus it is unlikely that the molecule is involved in metal sequestration in this case. In fact, when zinc concentration is reduced in the media, coproporphyrin production decreases by 65%. This may be due to the zinc dependence of several heme biosynthetic enzymes. However, rufomycin production, which requires heme enzymes, is unaffected by low Zn concentrations. Thus coproporphyrin III may play a role in zinc homeostasis, or the Zn^2+^ coproporphyrin complex is required for a currently unknown biological activity. *S. atratus* has been demonstrated to produce the potent siderophore nocardamine (desferrioxamine E) for iron sequestration which is likely to be the primary mode of iron acquisition in iron poor environments (Li et al., 2018). Some microorganisms have been shown to use coproporphyrin III as a chemical signal (Cleary et al., 2017) for example *Propionibacterium* spp. produces coproporphyrin III to induce aggregation and biofilm formation in *S. aureus* (Wollenberg et al., 2014). *Streptomyces atratus* has in fact been shown to be an effective biocontrol agent against fungi (Liang et al., 2016; Jung et al., 2018) and thus it is possible that coproporphyrin III is similarly used for intercellular or interspecies chemical signalling (Van Norman and Humans, 1974; Chengjie and Bernhard, 2015).

While the production of coproporphyrin III is intriguing, the additional production of heme degradation products biliverdin and bilirubin leads to further questions. The production of all three compounds appears to only be linked only by the fact that they are precursors and degradative products of heme biosynthesis respectively, and implies an upregulation of heme biosynthesis in the organism under rufomycin production conditions (Layer, 2021).

The biosynthetic pathway of the non-ribosomal peptide, rufomycin involves five heme-dependent enzymes (Ma et al., 2017; Tomita et al., 2017; Perez Ortiz et al., 2021). Heme-dependent enzymes particularly cytochrome P450s (CYPs) frequently play key roles in natural product biosynthetic pathways, where they introduce key functionalities *via* a range oxidative transformations, e.g., hydroxylation, C-C bond formation, epoxidation (Rudolf et al., 2017). As described above, the production of the biosynthetic precursor 3-nitro-L-tyrosine, is catalysed by the RufN and RufO nitrating system requiring a CYP and a heme dependent nitric oxide synthase (NOS; Ma et al., 2017; Tomita et al., 2017; Figure 1). We propose that nitric oxide production for this pathway may explain the production of biliverdin and bilirubin.

NOS/CYP catalysed aromatic nitration has been described in other streptomycetes (Barry et al., 2012; Dodani et al., 2014, 2016). The NOS, TxtD, and the CYP TxtE in the plant pathogen *Streptomyces scabies* biosynthesize 4-nitro-L-tryptophan (Barry et al., 2012), a precursor of the phytotoxin thaxtomin A. Reports on *Streptomyces scabies* indicate that nitric oxide produced during thaxtomin A biosynthesis diffuses out of the cell (Johnson et al., 2008). Nitric oxide is an important signalling molecule in plants thus NO produced by *S. scabies* is proposed to participate in host -pathogen interactions (Johnson et al., 2008). Our data indicate that like *S. scabies*, NO is detectable in *S. atratus* cells and may diffuse beyond the cell. *Streptomyces atratus* is not a known plant pathogen however, as nitric oxide has been shown to have many roles in bacteria from gene regulation, biofilm formation, lifestyle switching (Cleary et al., 2017), it may play a dual role as biosynthetic precursor and signalling molecule, perhaps in fungal or plant interactions in the soil.

Intracellular NO in *S. atratus* could contribute to oxidative and nitrosative stress. Bilirubin and biliverdin production have been shown to provide a mechanism to combat the deleterious effects of intracellular NO. The roles of biliverdin and bilirubin in combating oxidative and nitrosative stress in bacteria are poorly understood. Bilirubin is oxidatively unstable, reacting with ROS to produce bilirubin oxidation products (BOX) (Bulmer et al., 2018; Ritter et al., 2018). In eukaryotes, bilirubin has been indicated as a scavenger of nitric oxide and the reactive nitrating species, peroxynitrite (Ritter et al., 2018; Figure 2). Our data shows that we can inhibit NOS and thus NO production using a chemical inhibitor. The result is a reduction in rufomycin production which is expected by the reduction in production of a biosynthetic precursor, NO (Figure 2). Additionally, coproporphyrin III production is unaffected by NOS inhibition, indicating it has no role in protecting the cell against nitrosative stress. This is expected as coproporphyrin III has not previously been reported to have such a role. The results for biliverdin were however inconclusive. We observed, in general, reduced levels of biliverdin production when NO production was inhibited. However, it is difficult to quantify this effect due to the low levels of biliverdin produced and its instability. Further, experiments are required to unambiguously prove this link.

The genetic context of the biliverdin reductase gene (*bvr_satr_*), appears to be conserved in many *Streptomyces* strains, though not all *Streptomyces* genomes encode nitric oxide synthases to oxidatively produce NO. We note that F420-dependent biliverdin reductase (F-BVR) in *M. tuberculosis* appears to combat nitrosative stress resulting from exogenous ROS and NOS production. In the case of *M. tuberculosis*, biliverdin reductase promotes virulence by producing bilirubin as a defense mechanism against the oxidative attack of the immune system (Ahmed et al., 2016) and F420 negative *Mtb* mutant strains are sensitive to nitrosative stress (Gurumurthy et al., 2013).

The contexts of BVR encoding genes in *Streptomyces* and *Mtb* are very different, which is unsurprising given their different niches (Supplementary Figures 10, 11). Intriguingly, in *Streptomyces, F-br_star_* is preceded by a gene encoding a putative iron–sulfur cluster protein, frequently annotated in *Streptomyces* genomes as a Rieske non-heme oxygenase enzyme. Our analysis shows that the protein contains potential iron–sulfur cluster binding residues, but they do not constitute a Reiske iron–sulfur cluster conserved motif which would be found in the N-terminal domain (Supplementary Figure 12). The protein is also truncated and thus does not contain the non-heme iron binding domain to enable it to carry out Reiske type transformations, such as aromatic oxidation (Kweon et al., 2008). Thus, we conclude that this gene has been misannotated. Intriguingly, iron–sulfur cluster proteins are often involved in oxygen and nitric oxide sensing in bacteria (Sun et al., 2012; Mettert and Kiley, 2015; Hossain and Boon, 2017). This putative iron–sulfur protein does not have any homology to previously characterised oxygen/nitric oxide sensing proteins. Thus, it may represent a novel regulatory protein. Further extensive genetic and biochemical investigations are required to fully understand and elucidate its role.

In conclusion, we have identified the production of three tetrapyrroles, coproporphyrin III, biliverdin and bilirubin from the important antimycobacterial producing strain *S. atratus*. While we have proposed roles for all three molecules, further detailed studies are required to confirm these hypotheses. We propose that a highly conserved region of *Streptomyces* genomes indicates a cluster of genes responsible for dealing with oxidative and nitrosative stress. The importance of *Streptomyces* in the discovery and production of bioactive molecules (antibiotics and other therapeutics) as well as the key role they play in the soil, indicates a need for a better understanding of their metabolism and the roles of nitric oxide in this genus.

## Data availability statement

The original contributions presented in the study are included in the article/Supplementary material, further inquiries can be directed to the corresponding author.

## Author contributions

GP-O, JS, JP, and SB designed the experiments, GP-O, JS, DC, YD, and JP performed the experiments. GP-O, JS, DC, JP, YD, and SB analysed the data and wrote the manuscript. All authors contributed to the article and approved the submitted version.

## Funding

The Mexican National Council of Science and Technology (CONACyT)/Mexican Secretary of Energy (SENER) provided a scholarship to GP-O. Natural products work in the Barry lab has been funded by the MRC (MC_PC14105 v.2) and BBSRC (BB/P019811/1). KCL provided a PhD scholarship to JP.

## Conflict of interest

The authors declare that the research was conducted in the absence of any commercial or financial relationships that could be construed as a potential conflict of interest.

## Publisher’s note

All claims expressed in this article are solely those of the authors and do not necessarily represent those of their affiliated organizations, or those of the publisher, the editors and the reviewers. Any product that may be evaluated in this article, or claim that may be made by its manufacturer, is not guaranteed or endorsed by the publisher.
