# Supplementary material for: Production of copropophyrin III, biliverdin and bilirubin by the rufomycin producer, Streptomyces atratus
Source: Front Microbiol. 2023 Mar 16;14:1092166. doi: 10.3389/fmicb.2023.1092166 (PMC10060970; doi:10.3389/fmicb.2023.1092166)
Supplement: Supplementary file 1 [file Data_Sheet_1.pdf]

Supplementary Material

Contents

List of Suplementary Figures 1
List of Supplementary Tables 2
Supplementary Methods 3
Supplementary Figures and Tables 4
Supplementary References 21

List of Suplementary Figures

Supplementary Figure 1: *S. atratus* metabolites. 4

Supplementary Figure 2: Identification of Coproporphyrin III. 5

Supplementary Figure 3: ^{1}H NMR of coproporphyrin II and standard. 9

Supplementary Figure 4: Detection of bilirubin in *Streptomyces atratus* cultures. 10

Supplementary Figure 5: Structure and fluorescent properties of the NO probe 4-Amino-5-Methylamino-2',7'-Difluorofluorescein Diacetate (DAF-FM DA). 11

Supplementary Figure 6: Visualisation of NO production by *S. atratus* with DAF-FM DA.. 12

Supplementary Figure 7: Effect of L-NAME on *S. atratus* metabolite profile.. 13

Supplementary Figure 8: Effect of hemin on *S. atratus* metabolite profile.. 16

Supplementary Figure 9: Biliverdin producing heme oxygenase and biliverdin reductase in *S. atratus* DSM41673. 17

Supplementary Figure 10: Alignment of various gene clusters containing a F420-dependent biliverdin reductase.. 18

Supplementary Figure 11: Genetic context of characterized F420 Biliverdin reductase encoding gene (F-bvr, Rv2074, green), *M. tuberculosis* H37Rv (AL123456.3, 2323317-233288).. 19

Supplementary Figure 12: Alignment of putative conserved Fe2S2 in streptomyces species.. 20

Supplementary Figure 13: Production levels of rufomycins overtime in S. atratus, determined by HPLC analysis. 21

List of Supplementary Tables

Supplementary Table 1: The LC-MS mobile phase gradient profile used for the analysis of the crude extract 6

Supplementary Table 2: prep-HPLC mobile phase gradient profile used to analyse crude extract. 6

Supplementary Table 3: Analytical HPLC mobile phase gradient profile used for purification of metabolites 7

Supplementary Table 4: Mobile phase gradient profile used for the analytical separation of coproporphyrins 7

Supplementary Table 5: Mobile phase gradient profile used for HRMS analysis of coproporphyrins 8

Supplementary Table 6: Bacterial genomes used for comparison of biliverdin containing clusters. 14

Supplementary Table 7: Heme biosynthetic and degradation genes from relevant organisms. 15

Supplementary Methods

R2 + Yeast Extract medium (R2YE / R5 medium) for metabolite production(Shepherd et al., 2010)

- Sucrose……..... 51.5 g
- K_2SO_4 …......... 125 mg
- $MgCl_2 \cdot 6H_2O$……. 5 g
- Glucose…..……..... 5 g
- Casaminoacids…….. 50 mg
- Yeast extract ………... 2.5 g
- Bacto agar ………..…. 7.5 g
- Distilled water ……. 400 mL

Above solution was autoclaved. At time of use, the medium is remelted and the following autoclaved solutions were added and under sterile conditions before plates were poured:

- 5 mL of 0.5 % KH_2PO_4
- 40 mL of 3.7 % $CaCl_2 \cdot 2H_2O$
- 7.5 mL of 20 % L-proline
- 50 mL of 5.73 % TES buffer
- 2.5 mL of 1 M NaOH
- 663 mg of L-Leucine
- 1 mL of Trace Elements solution

Trace Elements solution: $ZnCl_2$ [40 mg L^{-1}], $FeCl_3 \cdot 6H_2O$ [200 mg L^{-1}], $CuCl_2 \cdot 2H_2O$ [10 mg L^{-1}], $MnCl_2 \cdot 4H_2O$ [10 mg L^{-1}], $Na_2B_4O_7 \cdot 10H_2O$ [10 mg L^{-1}] and $(NH_4)6Mo_7O_{24} \cdot 4H_2O$ [10 mg L^{-1}].

For the study of the effect of the reduction of metal ions, instead of using a Trace Elements solution, solutions of each ion were prepared, autoclaved and added separately.

The concentration of each solution was the same as the concentration of the respective ion in the Trace Elements solution.

Supplementary Figures and Tables

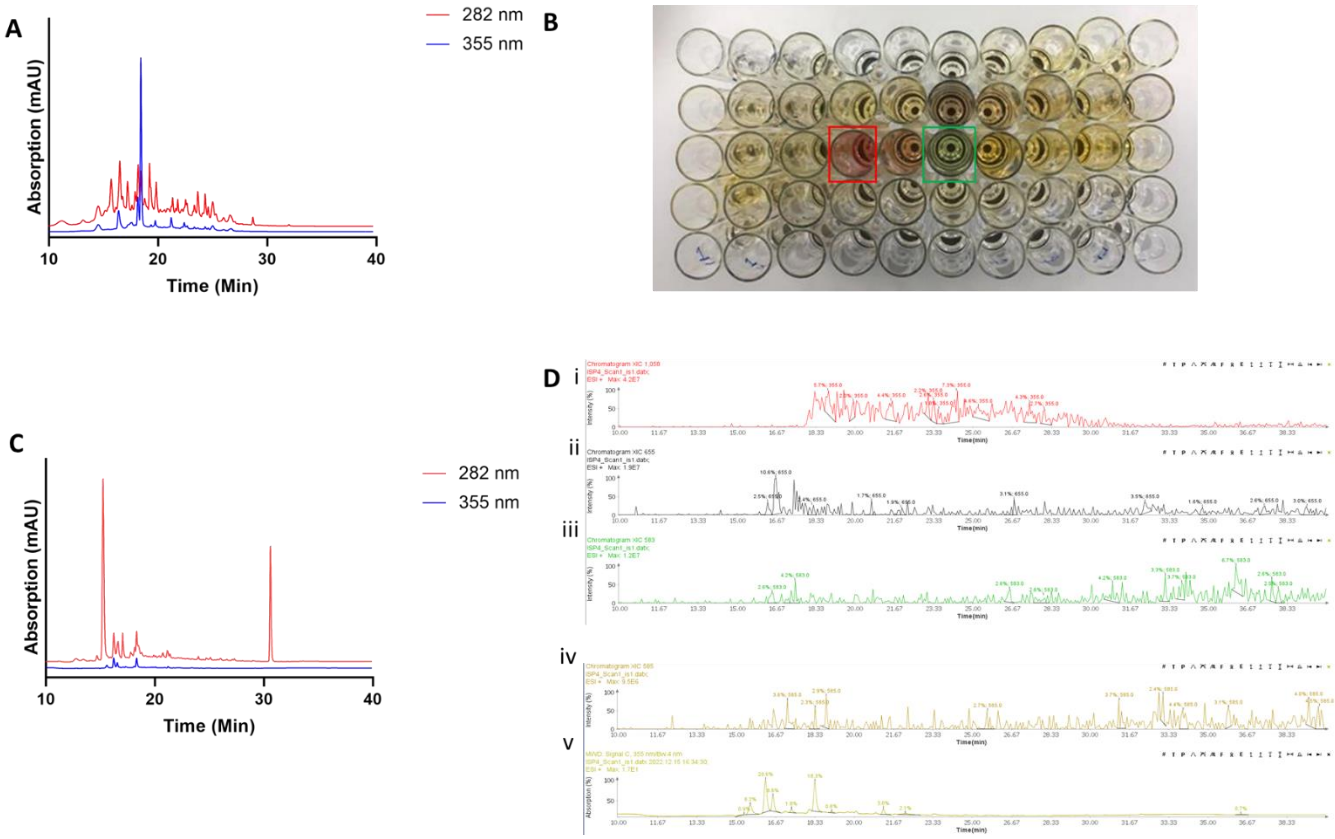

Supplementary Figure 1: ***S. atratus*** **metabolites. A)** Analytical LC trace of *S. atratus* grown on R5 media. B) Fractions collected from Preparative-HPLC of *S. atratus* culture extract grown on R5 media. A pink Fraction (fraction 1) containing Coproporphyrin III (red box) and a green fraction (fraction 2) containing biliverdin (green box) are clearly visible. C) Analytical LCMS trace of *S. atratus* grown on ISP4 media (used for spore production). D) Extracted ion chromatograms of target metabolites indicate production of rufomycins or target tetrapyrroles was detected. i. rufomycin C, ii. Coproporphryin III, iii. Biliverdin iv. Bilirubin, v. HPLC trace at 355 nm of crude extract.

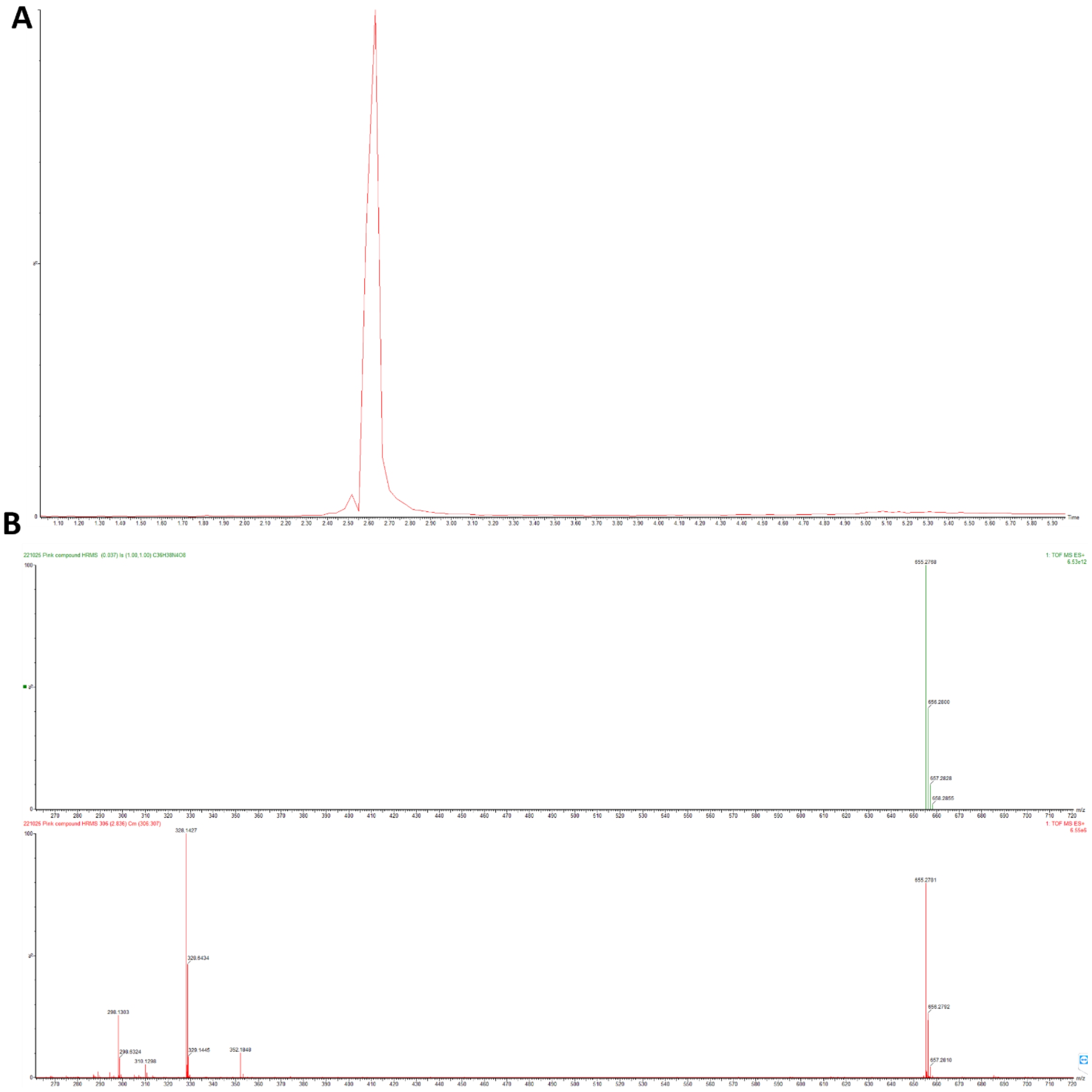

Supplementary Figure 2: Identification of Coproporphyrin III. A) Total Ion Chromatogram of purified coproporphyrin III from *S. atratus*. **B)** (top) Simulated mass spectrum for $[M+H]^+$ 655.2768, calculated for $C_{36}H_{39}N_4O_8$. (bottom) High resolution mass spectrum of *S. atratus* isolate, found $[M+H]^+$ 655.2781 (error 1.98ppm) and $[M+2H]^{2+}$ 328.1427. Data collected on ACQUITY UPLC BEH C8 column (1.7 µm, 2.1 x 50 mm). The detector was a Waters Xevo-G2-XS QTof with electrospray ionization source. The column temperature was 60 ºC. Mobile phase A was water + 0.1% formic acid; Mobile phase B was acetonitrile + 0.1% formic acid. The flow rate was 0.4 ml min^{-1}, with a 1 to 100 % Buffer B gradient in 5 minutes, followed by 1.5 minutes at 100 % Buffer B. The instrument was operated in positive mode full-scan with detection window set from 50 to 700 Da. For product ion scan, a collision energy ramp from 50 to 70 V was employed.

Supplementary Table 1: The LC-MS mobile phase gradient profile used for the analysis of the crude extract, 0.5 mL min^{-1} flow rate. FA= formic acid, ACN = acetonitrile

Time (min)	% Water +0.1% FA	% ACN +0.1% FA
0	100	0
1	80	20
30	65	35
33	10	90
35	10	90
36	100	0
42	100	0

Supplementary Table 2: prep-HPLC mobile phase gradient profile used to analyse crude extract, 20 mL min^{-1} flow rate. FA= formic acid, ACN = acetonitrile

Time (min)	% Water +0.1% FA	% ACN +0.1% FA
0	95	5
25	0	100
30	0	100
35	95	5
40	95	5

Supplementary Table 3: Analytical HPLC mobile phase gradient profile used for purification of metabolites, 0.5 mL min^{-1} flow rate FA= formic acid, ACN = acetonitrile

Time (min)	% Water +0.1% FA	%ACN +0.1% FA
0	100	0
1	90	10
30	30	70
31	10	90
35	10	90
36	100	0
42	100	0

Supplementary Table 4: Mobile phase gradient profile used for the analytical separation of coproporphyrins, 0.5 *mL min^{-1} flow rate TFA= trifluoracetic acid, ACN = acetonitrile.*

Time (min)	% Water +0.1% TFA	% Acetonitrile +0.1% TFA
0	100	0
1	80	20
30	65	35
33	10	90
35	10	90
36	100	0
42	100	0

Supplementary Table 5: Mobile phase gradient profile used for HRMS analysis of coproporphyrins, *0.5 mL min^{-1} flow rate. TFA= trifluoracetic acid, ACN = acetonitrile.*

Time (min)	% Water +0.1% FA	% Acetonitrile +0.1% FA
0	10	90
0.5	100	0
12.5	100	0
13	10	90

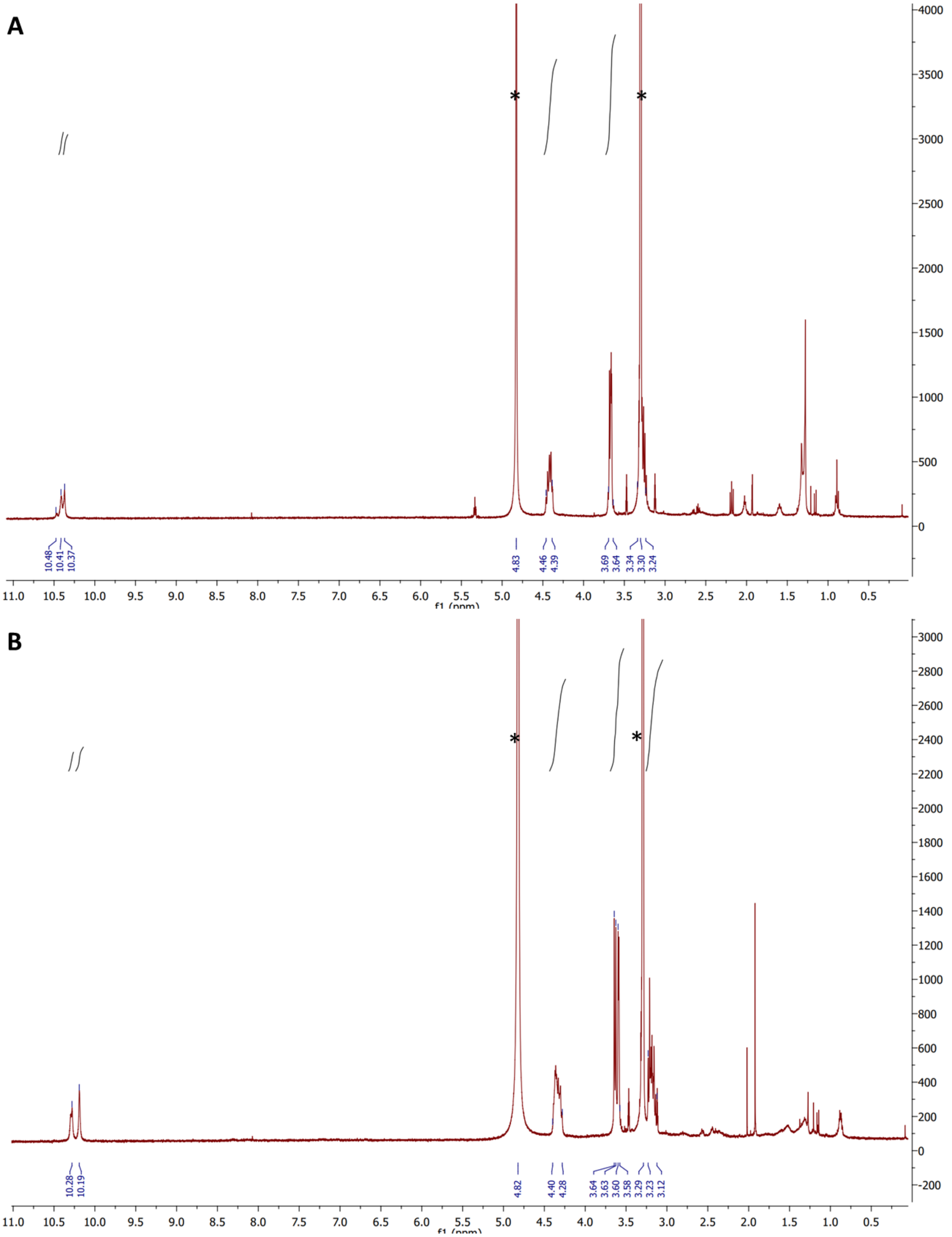

Supplementary Figure 3: ^{1}H NMR of coproporphyrin II and standard. A. ^{1}H NMR of coproporphyrin III standard. **B.** ^{1}H NMR of isolated and purified Coproporphyrin III (fraction 1). Solvent CD_3OD, 400MHz. *indicates residual solvent peaks, water (4.9ppm) and MeOH (3.3ppm). Differences in ppm shift likely due to pH/sample preparation differences.

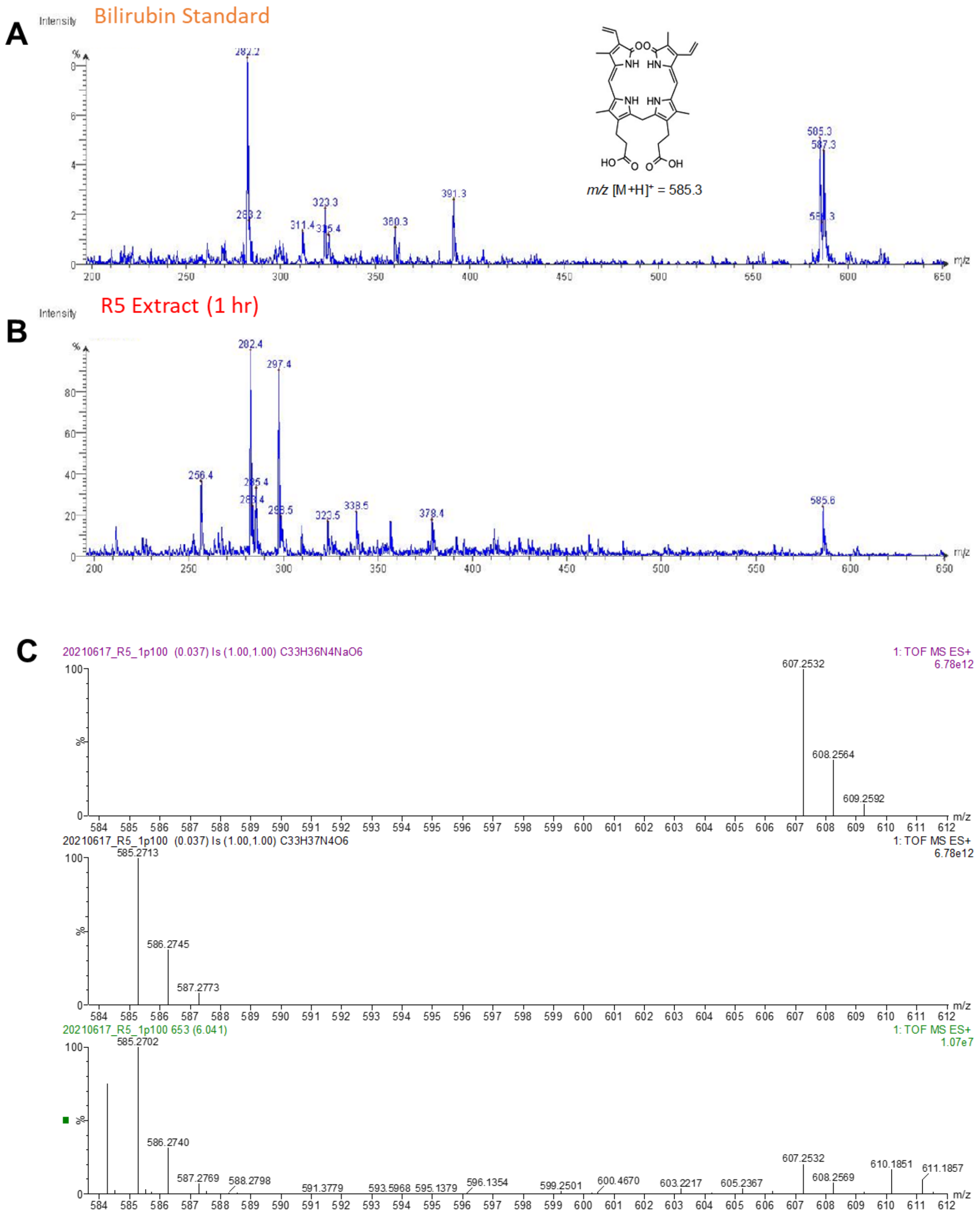

Supplementary Figure 4: Detection of bilirubin in *Streptomyces atratus* cultures. A. Mass spectrum of bilirubin standard (retention time, 33.1 min) **B.** The mass spectrum of the peak with the same retention time in the *S. atratus* R5 extract (analysed within 1hr). **C.** (top) Predicted mass spectrum calculated for $[M+Na]^+$ $C_{33}H_{36}N_4NaO_6$ m/z 607.2532. (middle) Predicted mass spectrum calculated for $[M+H]^+$ $C_{33}H_{37}N_4O_6$ m/z 585.2713. (bottom) High resolution mass spectrum of

bilirubin detected in culture extract within 1hr of extraction found $[M+Na]^+$ m/z 607.2532 (error 0 ppm) and $[M+H]^+$ 585.2702 (error -1.88 ppm).

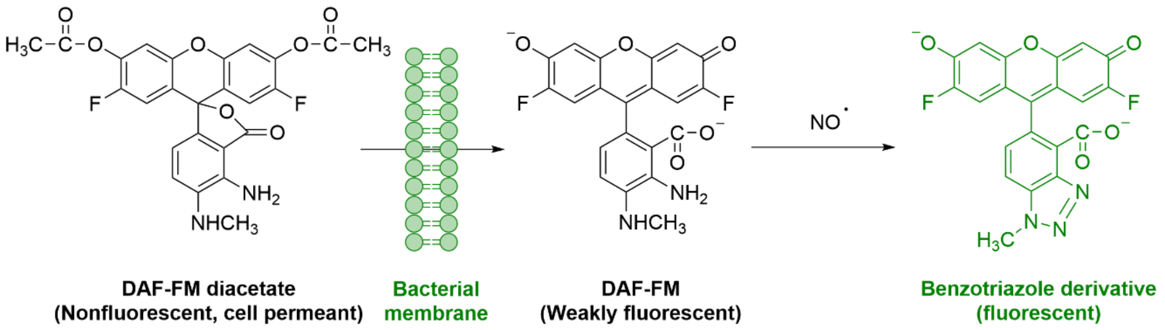

Supplementary Figure 5: Structure and fluorescent properties of the NO probe 4-Amino-5-Methylamino-2',7'-Difluorofluorescein Diacetate (DAF-FM DA). DA-FM enters the bacterial cell where intracellular esterases convert it to an ionic species which can react with nitric oxide to produce a fluorescent derivative (excitation 495 nm, emission 515 nm).

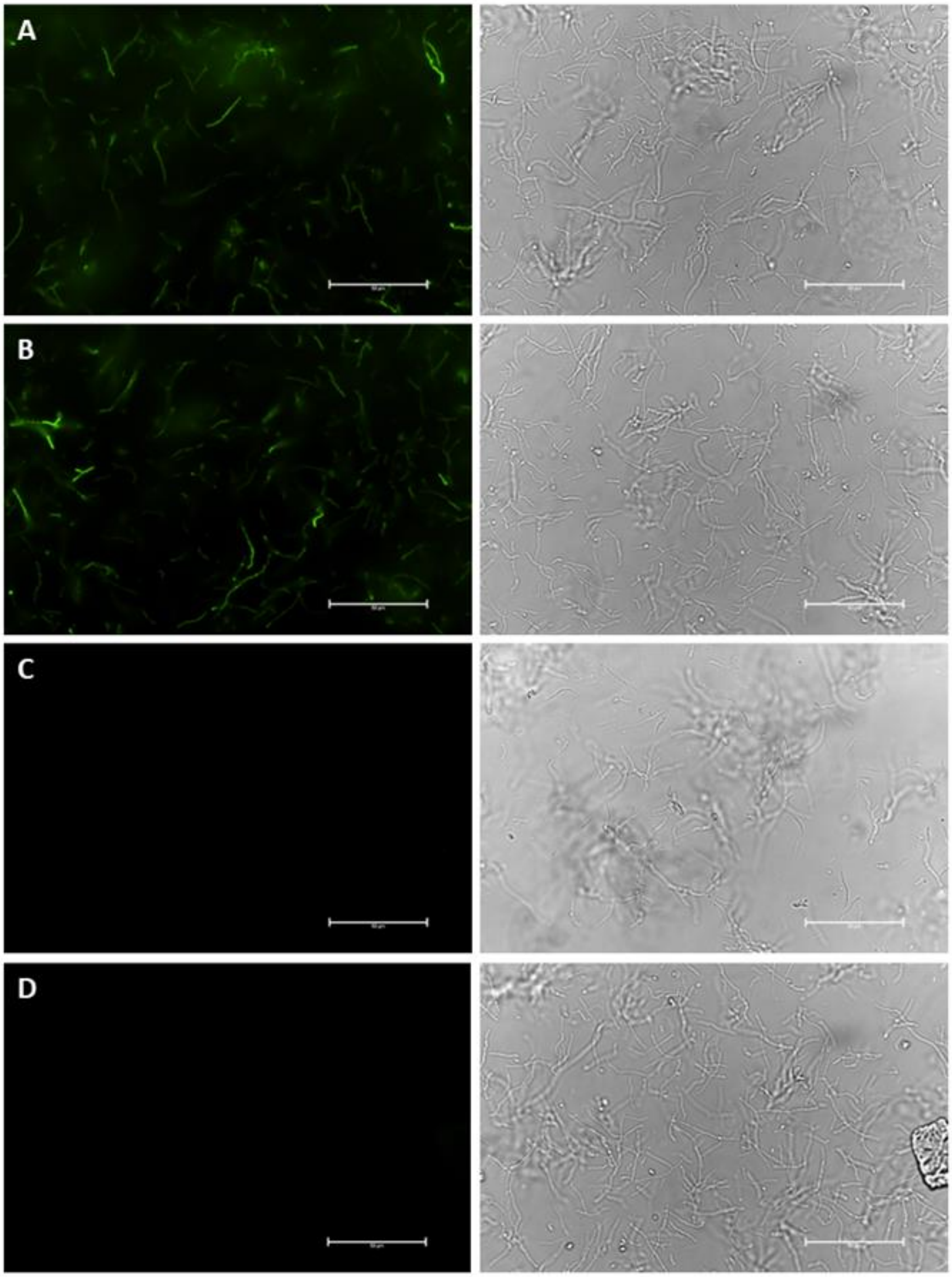

Supplementary Figure 6: Visualisation of NO production by *S. atratus* with DAF-FM DA. Visualisation of NO production by *S. atratus* with DAF-FM DA on a widefield microscope Eclipse Ti-2 inverted Nikon microscope equipped with a Plan Apo lambda 60x/1.40 Oil magnification. A-B show mycelia stained with DAF-FM DA. C-D are unstained controls. Fluorescence was excited with a pE-300ULTRA LED as a source light and collected with a Nikon FITC filter (465/495 emission, 515/555

absorption). Images were acquired with a Nikon DS-Qi2 sCMOS camera controlled with NIS elements software. Image analysis was done by NIS-Elements. Scale bars are 50 µM.

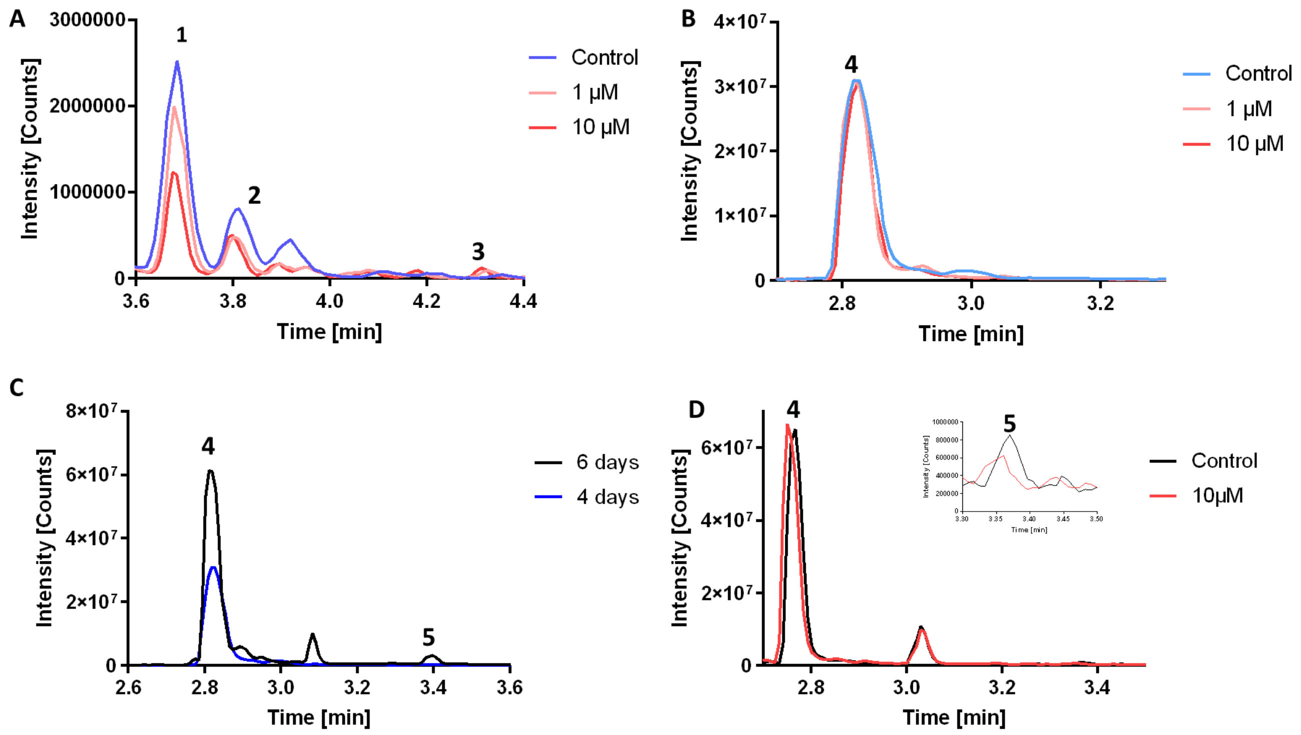

Supplementary Figure 7: Effect of L-NAME on *S. atratus* metabolite profile. A) HRMS analysis of *S. atratus* crude extract grown on R5 supplemented with L-NAME (0 µM (Control) 1 µM, 10 µM). Traces are extracted ion chromatograms including masses of rufomycins A **2**, B **3** and C **1** B) HRMS analysis of *S. atratus* crude extract grown on R5 supplemented with L-NAME (0 µM (Control) 1 µM, 10 µM). Traces are extracted ion chromatograms including mass of coproporphyrin III **4** **C)** HRMS analysis of *S. atratus* crude extract grown on R5 incubated for 4 days or 6 days. Traces are extracted ion chromatograms including mass of coproporphyrin III 4 and biliverdin **5.** **D)** HRMS analysis of *S. atratus* crude extract grown on R5 supplemented with L-NAME (0 µM (Control), 10 µM). (inset shows zoom of chromatogram 3.3min-3.5min). Traces are extracted ion chromatograms including mass of coproporphyrin III 4 and biliverdin **5.**

Actinobacteria Genome	NCBI Accession Number
Streptomyces sp PAMC26508	CP003990.1
Streptomyces fulvissimus NA06532	CP054926.1
Streptomyces atratus SCSIO ZH16	CP027306.1
Streptomyces griseus NBRC 13350	AP009493.1
Kitasatospora albolonga YIM 101047	CP020563.1
Streptomyces anulatus VUW1	CP080029.1
Streptomyces pratensis ATCC 33331	CP051486.1
Streptomyces venezuelae ATCC 15068	CP029194.1
Streptomyces atratus DSM41673 / JCM3386	GCA_014648655*

Supplementary Table 6: Bacterial genomes used for comparison of biliverdin containing clusters. **S. atratus* DSM41673/ JCM 3386 (GCA_014648655) shotgun sequence not assembled.

Gene Name	Gene Function	Organism	Accession Number
HMO_{satr}	Heme oxygenase	*S. atratus* DSM41673	WP_189277514
SfHO	Heme oxygenase	*S. fulvissimus* NA06532	SFUL_1847
BVR_{satr}	Biliverdin reductase	*S. atratus* DSM41673	WP_114245335
Rv2074	Biliverdin reductase	*M. tuberculosis* ATCC 25618	MTCY49.13
hemL	GSA-2,1-aminomutase	*S. atratus* DSM41673	WP_189281073
hemB	Porphobilinogen synthase	*S. atratus* DSM41673	WP_189281504
hemD	Uroporphyrinogen III synthase	*S. atratus* DSM41673	WP_189281502
hemC	Hydroxymethylbilane synthase	*S. atratus* DSM41673	WP_189281500
hemA	Glutamyl-tRNA reductasa	*S. atratus* DSM41673	WP_189281498
hemH	ferrochelatase	*S. atratus* DSM41673	WP_189275430
hemE	Uroporphyrinogen decarboxylase	*S. atratus* DSM41673	WP_189275954
hemY	Protoporphyrinogen oxidase	*S. atratus* DSM41673	WP_189275593
hemQ	Coproheme decarboxylase	*S. atratus* DSM41673	WP_114247285

Supplementary Table 7: Heme biosynthetic and degradation genes from relevant organisms. NCBI GeneIDs and their assigned putative function and organism of origin.

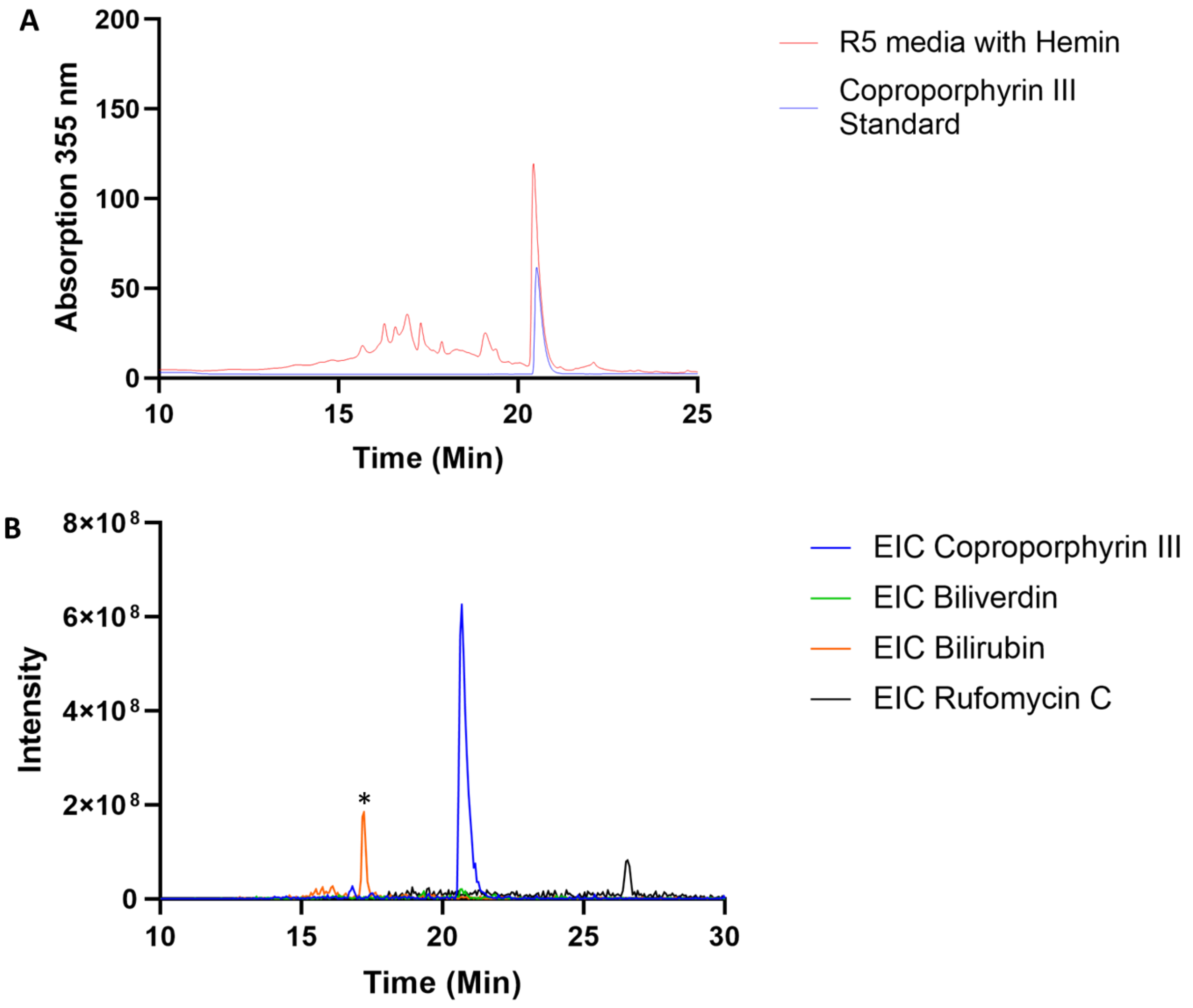

Supplementary Figure 8: Effect of hemin on *S. atratus* metabolite profile. Analysis of crude extract of *S. atratus* grown on R5 supplemented with hemin (10 nM). A) Trace at 355nm compared to coproporphyrin III standard. B) extracted ion chromatograms of LCMS analysis for mass of each compound of interest. No biliverdin was detected. no bilirubin was detected (* indicates peak with similar mass but different retention time to bilirubin).

```
S.flavissimus        MDATATVTPFSTLIRTASHEQHTEAETSTFMGDLLGGRLGVDAYTRYTEQLWFVYRALEE 60
S.atratus            LDATAAATPFSTLIRTASHEQHTEAETSTFMSDLLGGHLGVDAYTRYTEQLWFVYRALEE 60
                     :****:.************************.*****:**********************

S.flavissimus        GAEALRNDPVAGPFIQPELMRSTELERDLAHLRGENWREGLEPLPATAAYAARVTECART 120
S.atratus            GARALRNDPVAGPFIQPELMRTTELERDLAHLRGTGWREGLAPLPATAAYAARVAECART 120
                     **.******************:************ .***** ************:*****

S.flavissimus        WPAGYIAHHYTRYLGDLSGGQIIRDKAEKTWGFERKGDGVRFYVFEEIGNPAAFKRGYRE 180
S.atratus            WPAGYIAHHYTRYLGDLSGGQIIRDKAEKTWGFARKGDGVRFYVFEEISNPASFKRSYRE 180
                     ********************************* **************.***:***.***

S.flavissimus        LLDAVNADDLEKQRIVEECKRAFALNTAVFRELGEVFPLSA    221
S.atratus            LLDAVNADDLEKQRIVDECKQAFALNTAVFRELGEVFPLS-    220
                     ****************:***:*******************
```

```
M.tuberculosis       MAMVNTTTRLSDDALAFLSERHLAMLTTLRADNSPHVVAVGFTFDPKTHIARVITTGGSQ        60
S.atratus            --MAVDVTNPGPEYLAFWRARHVCTLTTPRPDGTPHVVPVGVTYDPGTRLARVITGKNTR        58
                       *.  .*. . : ***   **:. *** * *.:**** **.*:** *::*****  .::

M.tuberculosis       KAVNADRS----GLAVLSQVDGARWLSLEGRAAVNSDIDAVRDAELRYAQRY-RTPRPNP        115
S.atratus            KAANVLAAGPDGARVAVCQIDGRRWATLEGRATVRTEPEAVADAVRRYAERYERTPKPNP        118
                     **.*.   :     . ..:.*:** ** :*****:*.:: :** **   ***:** ***:***

M.tuberculosis       RRVVIEVQIERVLGSADLLD        135
S.atratus            DRIVIEIAVDRALGRA----        134
                      *:***: ::*.** *
```

Supplementary Figure 9: Biliverdin producing heme oxygenase and biliverdin reductase in *S. atratus* DSM41673. Biliverdin producing heme oxygenase bears high similarity (> 92.2% identity) with the previously described homologous protein of *S. flavissimus*. On the other hand, a biliverdin reductase protein identified in *S. atratus* bears high similarity (> 48.5% identity) with the F420-dependent biliverdin reductase characterized in *M. tuberculosis*. Accession number in Supplementary Table 7.

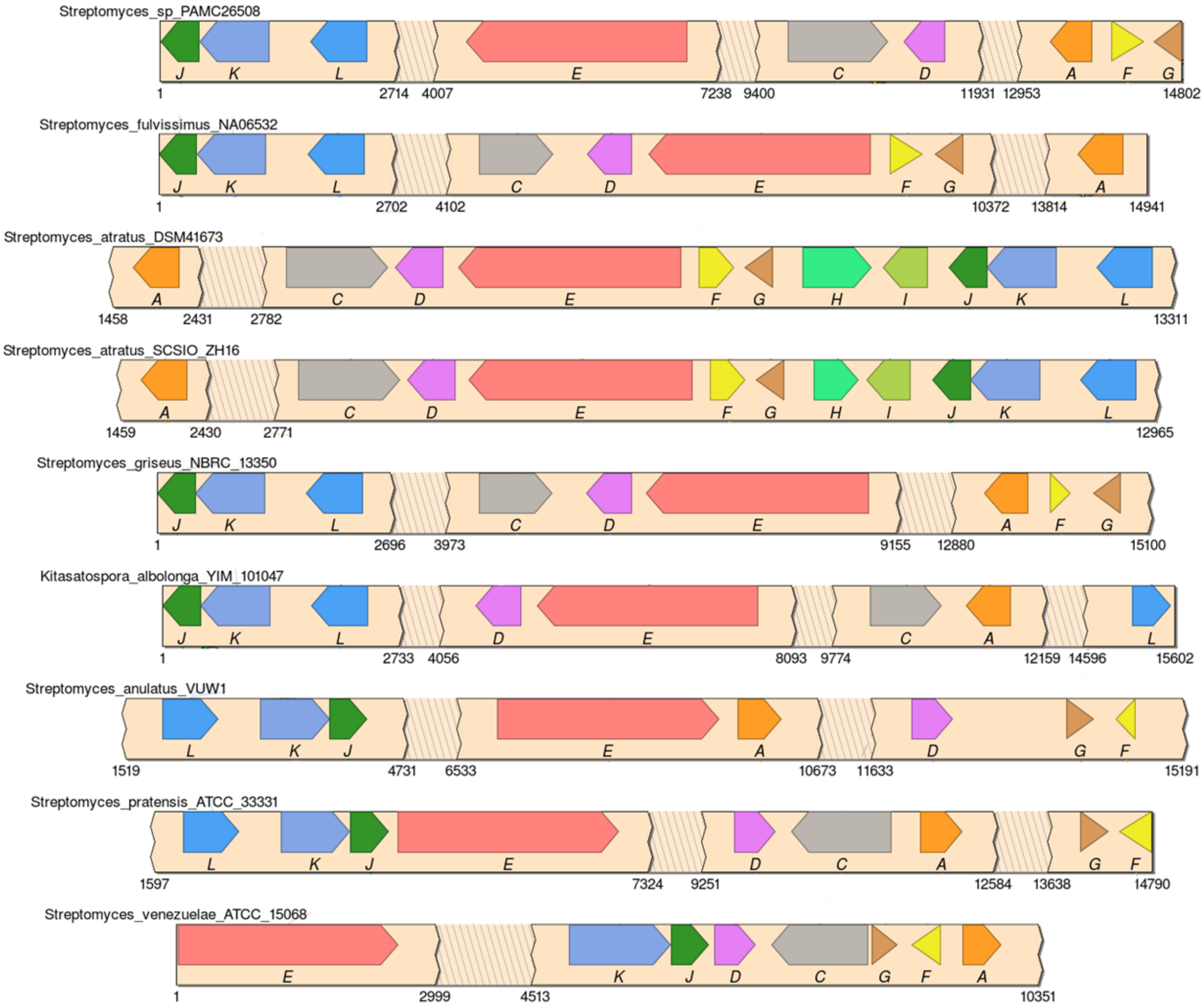

Supplementary Figure 10: Alignment of various gene clusters containing a F420-dependent biliverdin reductase. The F420-dependent biliverdin reductase containing gene cluster of *S. atratus* strains DSM41673 and SCSIO_ZH16 were aligned with seven species of actinobacteria. Coding regions are shown as arrows. Genes encoding similar products are displayed in the same colour. A) DUF2617 domain-containing protein, C) DapC, D) YbjN domain-containing protein, E) Chaperone ClpB, F) Biliverdin reductase, G) (2Fe-2S) binding protein, H) Helix-turn-helix transcriptional regulator, I) Hypothetical protein, J) Heat-shock protein HspR, K) Chaperone **DnaJ, L)** Nucleotide exchange factor GrpE. Image generated with Simple Synteny (https://www.dveltri.com/simplesynteny/).

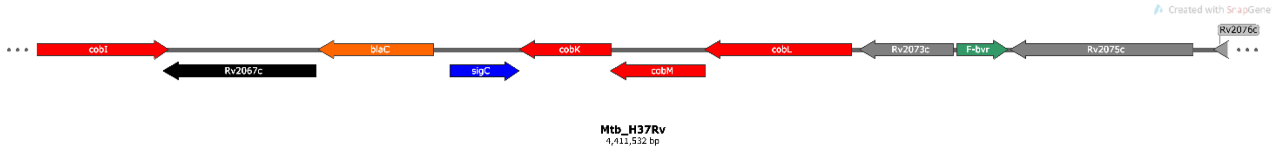

Supplementary Figure 11: Genetic context of characterized F420 Biliverdin reductase encoding gene (F-bvr, Rv2074, green), ***M. tuberculosis*** **H37Rv (AL123456.3, 2323317-233288).** *cobI, K, M, L* (cobalamin biosynthetic genes, red), *sigC* (transcription factor, blue), *blaC* (betalactamase, orange), Rv2067c (SAM dependent methyltransferase, black), Rv2073c (Short chain dehydrogenase, grey), Rv2075c (possible carbohydrate phospholipase, grey).

```
CLUSTAL O(1.2.4) multiple sequence alignment


Fe2S2           -------------------------------------TIPPDDRTFRREMATAYRSGW 21
SCO3662         ------------------------------------MTTPPDDRSFRREMATAYRSGW 22
CBG71301.1      ------------------------------------MTTPPDDRSFRREMATAYRSGW 22
1ULI            ----MTDVQCEPALAGRKPKWADADIAELVDERTGRLDPRIYTDEALYEQELERIFGRSW 56
3GZX            MSSTMKDTQEAP--VRWSRNWTPDAIRALVDQDNGKLDARIYADQDLYQLELERVFGRSW 58
                                                           *:  :. *:   :  .*

FE2S2           HFIDLVTAIPRCGDSLMVTLFGDPIVVAREEDDEVRAYRCLRKPRGA---------PQPV 72
SCO3662         HFIDLVTAIPHAGDSLMVTVFGEPVVVTLDEDGDVRAYRCLRRPRGA---------PQPV 73
CBG71301.1      HFIDLATAIPHSGDSLMVTVFGEPVVVTRDEDEDIRAYRCLRRPRGA---------PQPV 73
1ULI            LLMGHETQIPKAGDFMTNYMGEDPVMVVRQKNGEIRVFLNQCRHRGMRICRADGGNAKSF 116
3GZX            LMLGHETHIPKIGDYLTTYMGEDPVIMVRQKDQSIKVFLNQCRHRGMRIVRSDGGNAKAF 118
                 ::.  * **: ** :   :  :*:::. ::: .::.:    : **            : .

FE2S2           RCAIR----------------------------------------------YDMIFVNLDR 87
SCO3662         RCAIR----------------------------------------------YGMIFVNLDQ 88
CBG71301.1      RCAVR----------------------------------------------YGMVFVNLDQ 88
1ULI            TCSYHGWAYDTGGNLVSVPFEEQAFP-------GLRKEDWGPLQARVETYKGLIFANWDA 169
3GZX            TCTYHGWAYDIAGNLVNVPFEKEAFCDKKEGDCGFDKADWGPLQARVETYKGLVFANWDP 178
                 *: :                                              .::*.* *

FE2S2           RDHQLFEPETI---------SATPRS--A------------------------------- 105
SCO3662         RDHRLEQPEIPEVR----TISATPRS--A------------------------------- 111
CBG71301.1      RDHRLAEPETPAST----AVTATPRS--A------------------------------- 111
1ULI            DAPDLDTYLGEAKFYMDHMLDRTEAGTEAIPGIQKWVIPCNWKFAAEQFCSDMYHAGTTS 229
3GZX            EAPDLKTYLSDAMPYMDVMLDRTEAGTEAIGGIQKWVIPCNWKFAAEQFCSDMYHAGTMS 238
                    *                  *  .  *

FE2S2           ------------------------------------------------------------ 105
SCO3662         ------------------------------------------------------------ 111
CBG71301.1      ------------------------------------------------------------ 111
1ULI            HLSGILAGLPDGVDLSELAPPTEGIQYRATWGGHGSGFYIGDPNLLLAIMGPKVTEYWTQ 289
3GZX            HLSGVLAGLPPEMDLTQIQLSKNGNQFRSAWGGHGAGWFINDSSILLSVVGPKITQYWTQ 298


FE2S2           ------------------------------------------------------------ 105
SCO3662         ------------------------------------------------------------ 111
CBG71301.1      ------------------------------------------------------------ 111
1ULI            GPAAEKASERLGSTERGQQLMAQHMTIFPTCSFLPGINTIRAWHPRGPNEIEVWAFTVVD 349
3GZX            GPAAEKAARRVPQLP-ILDMFGQHMTVFPTCSFLPGINTIRTWHPRGPNEVEVWAFVLVD 357


FE2S2           ------------------------------------------------------------ 105
SCO3662         ------------------------------------------------------------ 111
CBG71301.1      ------------------------------------------------------------ 111
1ULI            ADAPEEMKEEYRQQTLRTFSAGGVFEQDDGENWVEIQQVLRGHKARSRPFNAEMGLGQTD 409
3GZX            ADAPEDIKEEFRLQNIRTFNAGGVFEQDDGENWVEIQRVMRGHKAKSTSLCAKMGLNVPN 417


FE2S2           ---------------------------------------------------- 105
SCO3662         ---------------------------------------------------- 111
CBG71301.1      ---------------------------------------------------- 111
1ULI            SDNPDYPGTISYVYSEEAARGLYTQWVRMMTSPDWAALDATRPAVSESTHT 460
3GZX            KNNPAYPGKTAYVYAEEAARGMYHHWSRMMSEPSWDTLKP----------- 457
```

Supplementary Figure 12: Alignment of putative conserved (2Fe-2S) in streptomyces species. Alignment of putative conserved *Streptomyces* (2Fe-2S) (WP_093899390) binding protein from *S. atratus* with homologous proteins from the model Streptomycete, *S. coeilcolor* (SCO3663) and plant

pathogen, *Streptomyces scabiei* 87.22 (CBG71301) and two structurally characterized Rieske proteins Biphenyl Dioxygnease, *Rhodococcus* sp. strain RHA1 (PDB: 1ULI) and Biphenyl Dioxygenase, *Comamonas testosteroni* Sp. Strain B-356 (PDB: 3GZX) from a blastP search against the PDB. Residues highlighted in green are conserved non-heme iron binding site and essential for catalysis. Residues in pink bind the (2Fe-2S) Rieske cluster. Residues in yellow represent potential (2Fe-2S) cluster binding residues. It is clear from the alignment that the non-heme oxygenase domain is missing.

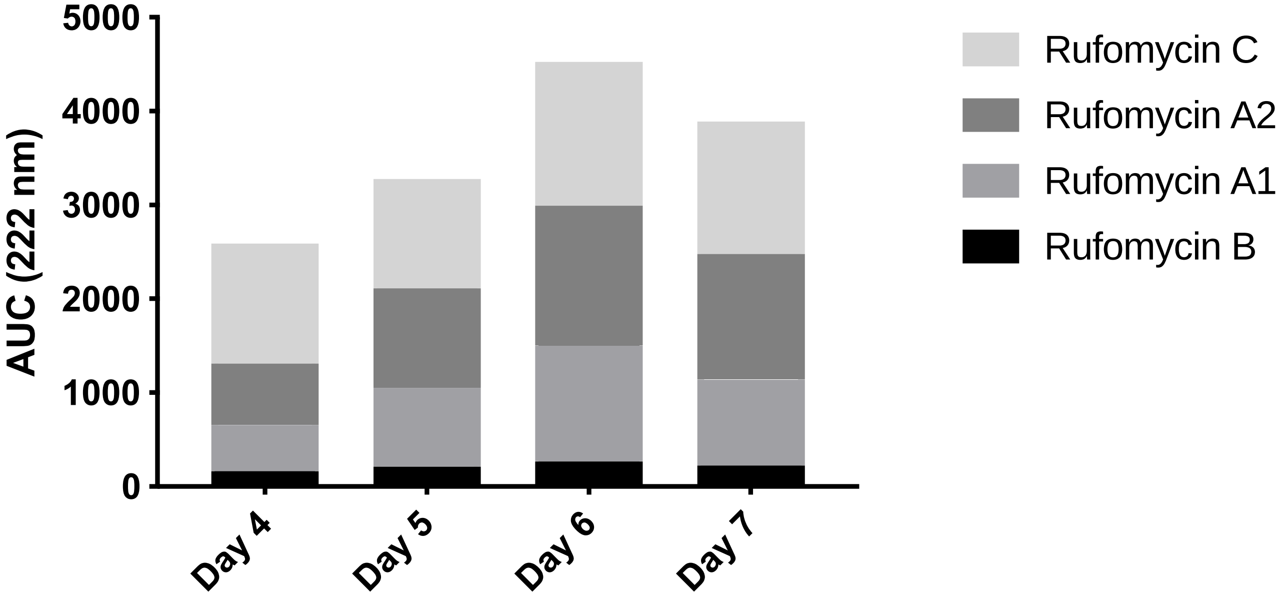

Supplementary Figure 13: Production levels of rufomycins overtime in *S. atratus*, determined by HPLC analysis.

Supplementary References

Shepherd, M.D., Kharel, M.K., Bosserman, M.A., and Rohr, J. (2010). Laboratory maintenance of Streptomyces species. *Curr Protoc Microbiol* Chapter 10**,** Unit 10E 11. doi: 10.1002/9780471729259.mc10e01s18.
